# Analysis of Genome Sequences from Plant Pathogenic *Rhodococcus* Reveals Genetic Novelties in Virulence Loci

**DOI:** 10.1371/journal.pone.0101996

**Published:** 2014-07-10

**Authors:** Allison L. Creason, Olivier M. Vandeputte, Elizabeth A. Savory, Edward W. Davis, Melodie L. Putnam, Erdong Hu, David Swader-Hines, Adeline Mol, Marie Baucher, Els Prinsen, Magdalena Zdanowska, Scott A. Givan, Mondher El Jaziri, Joyce E. Loper, Taifo Mahmud, Jeff H. Chang

**Affiliations:** 1 Department of Botany and Plant Pathology, Oregon State University, Corvallis, Oregon, United States of America; 2 Molecular and Cellular Biology Program, Oregon State University, Corvallis, Oregon, United States of America; 3 Laboratoire de Biotechnologie Vegetale, Universite Libre de Bruxelles, Gosselies, Belgium; 4 University of Antwerp, Department of Biology, Laboratory of Plant Growth and Development, Antwerp, Belgium; 5 Informatics Research Core Facility, University of Missouri, Columbia, Missouri, United States of America; 6 United States Department of Agriculture, Agricultural Research Service, Horticultural Crops Research Laboratory, Corvallis, Oregon, United States of America; 7 Department of Pharmaceutical Sciences, Oregon State University, Corvallis, Oregon, United States of America; 8 Center for Genome Research and Biocomputing, Oregon State University, Corvallis, Oregon, United States of America; University of the West of England, United Kingdom

## Abstract

Members of Gram-positive Actinobacteria cause economically important diseases to plants. Within the *Rhodococcus* genus, some members can cause growth deformities and persist as pathogens on a wide range of host plants. The current model predicts that phytopathogenic isolates require a cluster of three loci present on a linear plasmid, with the *fas* operon central to virulence. The Fas proteins synthesize, modify, and activate a mixture of growth regulating cytokinins, which cause a hormonal imbalance in plants, resulting in abnormal growth. We sequenced and compared the genomes of 20 isolates of *Rhodococcus* to gain insights into the mechanisms and evolution of virulence in these bacteria. Horizontal gene transfer was identified as critical but limited in the scale of virulence evolution, as few loci are conserved and exclusive to phytopathogenic isolates. Although the *fas* operon is present in most phytopathogenic isolates, it is absent from phytopathogenic isolate A21d2. Instead, this isolate has a horizontally acquired gene chimera that encodes a novel fusion protein with isopentyltransferase and phosphoribohydrolase domains, predicted to be capable of catalyzing and activating cytokinins, respectively. Cytokinin profiling of the archetypal D188 isolate revealed only one activate cytokinin type that was specifically synthesized in a *fas*-dependent manner. These results suggest that only the isopentenyladenine cytokinin type is synthesized and necessary for *Rhodococcus* phytopathogenicity, which is not consistent with the extant model stating that a mixture of cytokinins is necessary for *Rhodococcus* to cause leafy gall symptoms. In all, data indicate that only four horizontally acquired functions are sufficient to confer the trait of phytopathogenicity to members of the genetically diverse clade of *Rhodococcus*.

## Introduction

Plant pathogenic bacteria employ an array of molecules to dampen immunity, alter plant physiological responses, and mimic plant hormones to modulate host-signaling pathways [1], [2]. Collectively and often coordinately, the virulence molecules manipulate host cells to give pathogens access to host tissues and resources as well as facilitate egress and spread. Because of the intimate interactions of these pathogen-synthesized molecules with host cells, virulence genes are subject to strong selective pressures and are often dynamic, exhibiting patterns of high genetic diversity [3]. Horizontal gene transfer (HGT) is one mechanism that contributes to evolutionary dynamism, as virulence genes are often found on plasmids, associated with mobile genetic elements, and/or located within large stretches of genomic islands that have signatures indicative of HGT [4]. Pathoadaptation, a process whereby mutations modify traits and improve virulence, has been implicated as another mechanism in the evolution of pathogenicity [5], [6].

Actinobacteria is one of the largest taxonomical units within the domain Bacteria and its members inhabit a diversity of ecosystems. A very small number of genera within Actinobacteria have members that are pathogenic to plants. *Rhodococcus* are non-spore forming, non-motile, mycolic acid-containing bacteria within Actinobacteria [7]. Its members are best known as environmental bacteria with a wide range of catabolic functions and large genomes ranging from ∼4 megabases (Mb) to ∼9 Mb [8]. *Rhodococcus fascians* is the first species in the genus characterized as a plant pathogen and it infects plants in an unusual manner [9], [10]. *R. fascians* grows epiphytically on the surface of leaves. During the transition to an endophyte, the pathogen breaches the host cuticle, collapses the epidermal layer, and forms ingression sites beneath epiphytic colonies [11]. The bacterium then grows inside the host tissue and provokes cell differentiation and *de novo* organogenesis, resulting in proliferations and abnormal growths called witches’ brooms or leafy galls [12]. *Rhodococcus* is a persistent pathogen and can remain associated with the plant throughout its life [13]. Its host range is exceedingly large and includes more than 120 species representing both monocots and dicots, herbaceous and woody plants [12].

**Figure 1:**
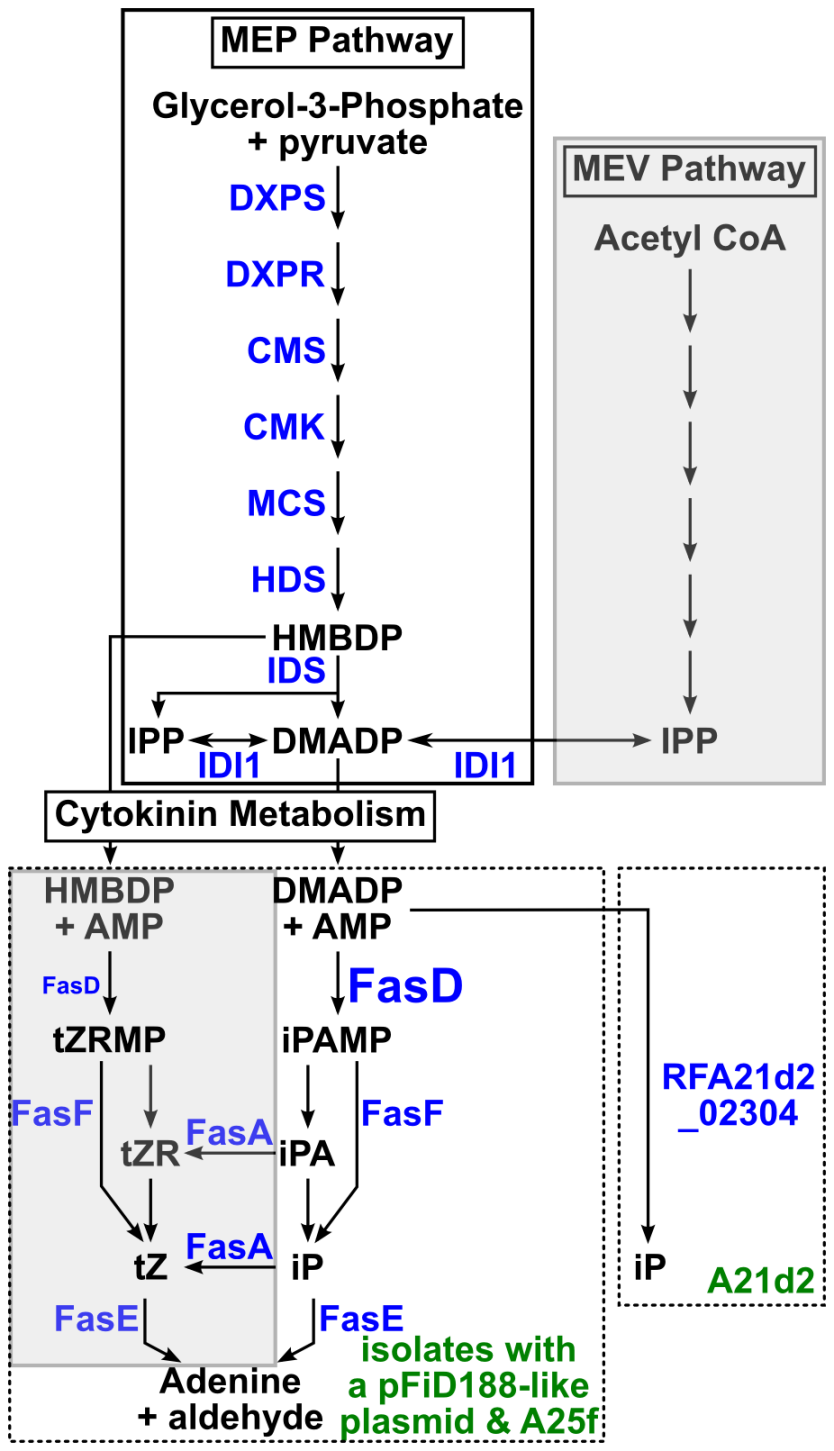
Predicted pathway of cytokinin metabolism in phytopathogenic *Rhodococcus*. Pathways for the biosynthesis of cytokinin precursors are presented within boxes with solid lines. Abbreviations: MEP = methylerythritol phosphate; HMBDP = (E)-4-hydroxy-3-methyl-but-2-enyl diphosphate; DMADP = dimethylallyl diphosphate; IPP = isopentenyl diphosphate; MEV = mevalonate. Abbreviations in blue indicate enzymes with homologs encoded by the 20 isolates of *Rhodococcus*: DXPS = Deoxyxylulose 5-phosphate synthase; DXPR = DXP reductiosomerase; CMS = 4-diphosphocytidyl-2C-methyl-D-erythirol synthase; CMS = 4-(cytidine 5′-diphospho)-2-C-methyl-D-erythritol kinase; MCS = 2-C-methyl-D-erythritol 2,4-cyclodiphosphate synthase; (HDS = hydroxy-2-methyl-2-(E)-butenyl 4-diphosphate synthase; IDS = hydroxy-2-methyl-2-(E)-butenyl 4-diphosphate reductase; IDI1 = isopentenyl diphosphate isomerase. The predicted cytokinin metabolism steps are presented within boxes with dotted lines. Left = products of the Fas operon (the difference in sizes of FasD symbolizes *in vitro* substrate preference). FasD is an isopentenyltransferase that transfers an isoprenoid moiety to adenine; FasA is a homolog of P450-type cytochrome monooxygenases that hydroxylate the isopentenyl side chain to produce trans-zeatin; FasF removes the ribose 5′-monophosphate and releases the corresponding activate free base cytokinin; FasE is a cytokinin oxidase/dehydrogenase that degrades and inactivates cytokinins. The functions for FasB and FasC (not shown) are not known, but are suggested to be accessory proteins that provide energy for cytokinin metabolism. See associated text for references. Right = predicted pathway by a novel protein fusion encoded in isolate A21d2. RFA21d2_02304 is predicted to encode a fusion protein with FasD- and FasF-like functions. Abbreviations: iPAMP = isopentenyladenine ribotide; tZRMP = trans-zeatin ribotide; tZR = trans-zeatin riboside; tZ = trans-zeatin; iP = isopentenyladenine; iPA = isopentenyladenosine. Shaded boxes = not supported by results of this study.

Phytopathogenicity of *R. fascians* D188 requires three virulence loci clustered on the conjugative linear plasmid, pFiD188 [14]–[19]. The *fasA-F* operon is the primary virulence operon and is implicated in the synthesis and modification of cytokinins, a class of plant growth regulating hormones (Fig. 1; [15], [18], [20]). The collective functions of the Fas proteins are hypothesized to be necessary for the pathogen to synthesize a mixture of cytokinins to upset homeostatic levels and cause and maintain leafy gall disease symptoms [10]. FasD is an isopentenyltransferase (IPT) and the key enzyme that transfers an isoprenoid moiety to adenine, the limiting step in cytokinin biosynthesis [14], [21]. A loss-of-function *fasD* mutant is non-pathogenic [14]. FasF is a homolog of LONELY GUY (LOG; a phosphoribohydrolase) of plants and functions to release activated cytokinins from their riboside forms [18], [22]. FasA is predicted to produce trans-zeatin (tZ)-types of cytokinins that are hypothesized to be important constituents of the bacterial-synthesized mixture of cytokinins [18], [20], [23]. The second locus is *fasR*, which encodes a predicted member of the AraC-like transcriptional regulatory protein family that is hypothesized to indirectly influence the transcription of *fasA-F* [10], [16], [18]. Finally, the *att* locus is also necessary for full virulence of *R. fascians* D188 [17]. The translated sequences for some of the *att* genes are homologous to antibiotic biosynthesis enzymes and thus predicted to be involved in secondary metabolism, though the specific metabolite(s) has yet to be identified.

Because of the absence of genomic resources and the focus on a single isolate, the evolution and contribution of other functions in the virulence of phytopathogenic *Rhodococcus* are poorly understood. However, insights into virulence evolution may be derived from characterizations of the *Rhodococcus equi* genome sequence [24]. *R. equi* infects mammals and is the only other species of this genus that is well documented as being pathogenic [25]. Comparisons of the *R. equi* 103S genome sequence to those of environmental species of *Rhodococcus* revealed little evidence for large-scale acquisition of niche-adaptation genes by HGT and instead suggested that pathogenicity of *R. equi* evolved through a limited number of key acquisition events coupled with co-option of genes core to *Rhodococcus* [24].

Here, we report the sequence and characterization of the genomes of 20 isolates of *Rhodococcus*. Our data indicate that HGT is important in virulence evolution but only four functions need to be acquired by members of *Rhodococcus* to gain the trait of phytopathogenicity. A striking discovery was made in isolate A21d2. This phytopathogenic isolate lacks the *fas* operon, which is replaced by a horizontally acquired and novel gene chimera. The protein fusion is predicted to be sufficient for the minimal functions of cytokinin catalysis and activation, typically provided by *fasD* and *fasF* of the *fas* operon. The absence of two-thirds of the *fas* operon from A21d2 is not consistent with the cytokinin mixture model. We profiled cytokinins in the wild type isolate D188 and its mutants, Δ*fasD* and ΔpFiD188, and could detect only one active cytokinin type that was synthesized in a *fasD*-dependent manner. Therefore, only one cytokinin type appears necessary for phytopathogenicity.

## Results

### Twenty *Rhodococcus* isolates were selected for genome sequencing

**Figure 2:**
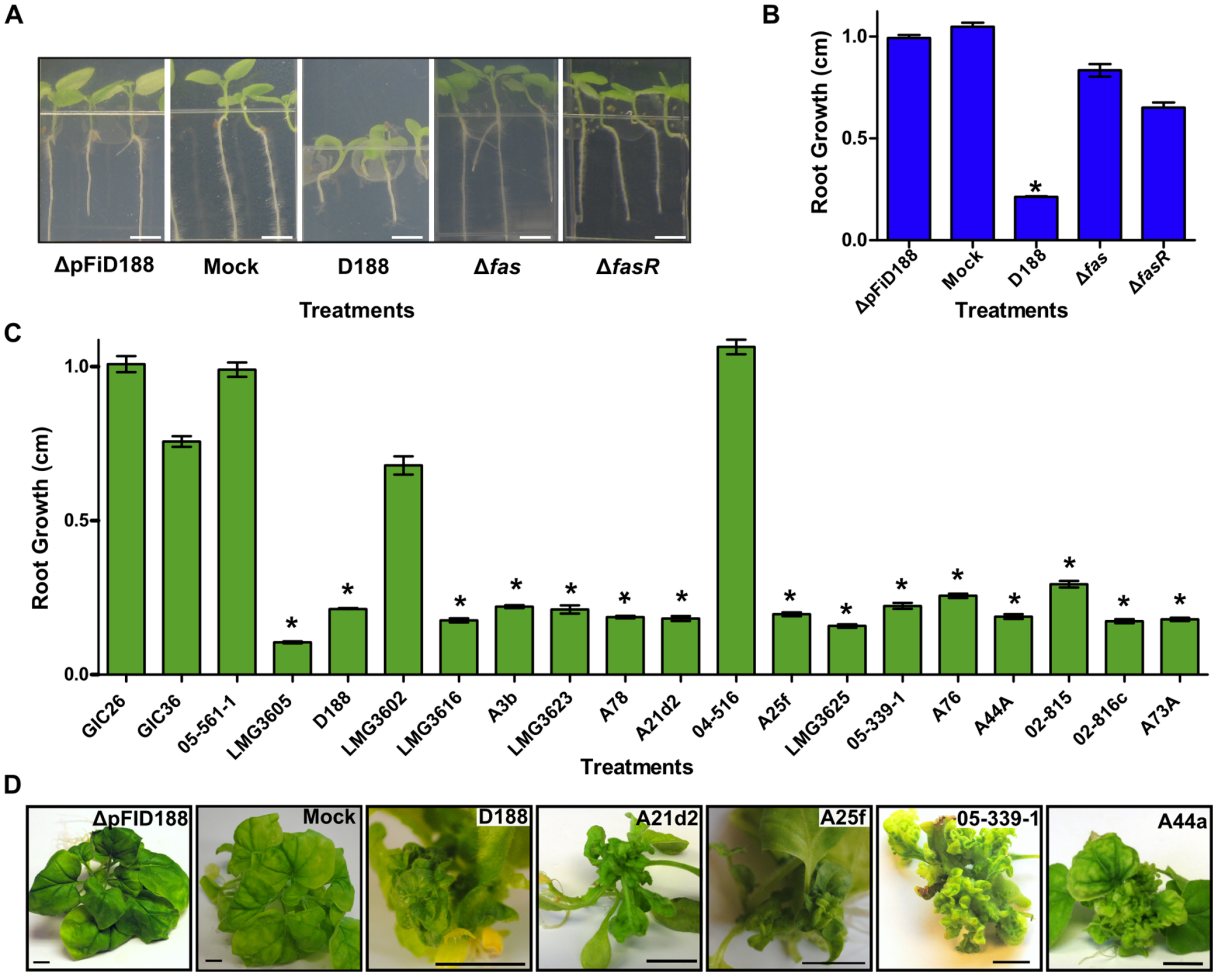
Sequenced isolates of *Rhodococcus* vary in phytopathogenicity. (A) Inhibition of *N. benthamiana* root growth by phytopathogenic *Rhodococcus*. Seedlings were independently inoculated with D188, its genetic variants, or 10 mM MgCl_2_ buffer (mock). Photographs were taken at 7 days after inoculation; scale bar = 0.25 cm. (B and C) Average root growth of *N. benthamiana* seedlings infected with D188 and its genetic variants (B) or with *Rhodococcus* isolates (C). Root lengths were measured (cm) and averaged. Error bars indicate standard error of the mean (SEM); *significant (p-value threshold ≤0.01). All treatments were repeated at least three times with similar results. (D) Isolates of *Rhodococcus* cause leafy gall disease. *N. benthamiana* was infected with the ΔpFiD188 strain of D188, mock, or members that represent the diversity of the clade. The black scale bar = 1 cm.

To quantify the virulence of the 20 selected isolates, we measured their effects on root growth of *Nicotiana benthamiana* seedlings [13]. First, we used wild type D188 and key mutants previously shown to be non-pathogenic or compromised in virulence to standardize the root inhibition assay (Figs. 2A and 2B). Of the 20 isolates, 15 isolates significantly inhibited root growth of *N. benthamiana* (Fig. 2C). The isolates we selected represent multiple clades of *Rhodococcus* (Creason and Chang, data not shown). Therefore, isolates, representing the genetic diversity of the sequenced samples, were tested and demonstrated to cause leafy galls to *N. benthamiana* (Fig. 2D).

A minimum of 17.5 million reads was generated for each genome sequence (Table 1). Isolates A44a and D188 were selected as references and were more deeply sequenced using hybrid approaches. The reads were processed and independently *de novo* assembled for each genome. The number of scaffolds ranged from 9–50. Isolate A44a had the fewest scaffolds, which we attribute to the use of mate pair sequencing. The A44a assembly had two scaffolds that corresponded to its linear and circular plasmids and seven for the chromosome. Based on the finished genomes of *R. jostii* RHA1 and *R. equi*, we infer that A44a has four rRNA-encoding loci that could account for three of the physical gaps in the sequence. The use of 454 reads helped reduce the number of scaffolds for D188 when assembled using Illumina reads alone, but relative to other assemblies, was not effective in dramatically improving the quality of the assemblies. All 20 sequenced isolates have a high GC% of around 64%. The average genome size and number of coding sequences (CDSs) for all 20 isolates was estimated at 5.8 Mb and 5,475, respectively.

**Table 1:** Summary statistics of *Rhodococcus* draft genome assemblies.

Isolate*	Pathogen¥	# usable reads	# scaffolds†	∼Size (Mb)	GC%	# CDSs	# tRNAs	Linear plasmid¥
GIC26	NO	18,924,378	49	5.3	64.45	5022	48	NO
GIC36	NO	18,778,318	46	5.6	64.48	5255	52	NO
05-561-1	NO	19,807,810	30	5.6	64.49	5303	50	NO
LMG3605	YES	19,206,754	27	5.3	64.53	4966	51	YES
*D188*	YES	65,408,710§	49	5.4	64.56	5125	51	YES
LMG3602	NO	18,978,318	25	5.4	64.50	5124	50	NO
LMG3616	YES	19,617,964	42	5.8	64.33	5486	51	YES
A3b	YES	20,464,110	34	6.0	64.21	5793	51	YES
**LMG3623**	YES	20,002,692	30	5.8	64.38	5426	48	YES
A78	YES	22,374,616	41	6.0	64.33	5684	50	YES
A21d2	YES	20,072,238	30	6.0	64.10	5626	54	NO
04-516	NO	19,136,324	23	5.8	64.20	5465	52	NO
A25f	YES	20,556,130	17	5.9	64.13	5544	50	NO
LMG3625	YES	17,750,698	17	5.9	64.10	5754	56	YES
05-339-1	YES	20,611,418	22	5.7	64.71	5449	54	YES
A76	YES	18,608,398	29	6.0	64.56	5720	53	YES
*A44a*	YES	87,282,290§	9	5.9	64.47	5584	54	YES
02-815	YES	22,244,434	30	6.2	64.28	5819	55	YES
02-816c	YES	23,697,112	45	6.1	64.52	5843	58	YES
A73a	YES	23,823,844	23	5.9	64.37	5501	56	YES

*Isolates designated with LMG were obtained from Belgium co-ordinated collection of micro-organisms (BCCM); GIC isolates are from a Greenland glacial ice core; all remaining isolates except D188, were obtained from diseased plants submitted to the Oregon State University (OSU) Plant Clinic. *Italicized* isolates = first sequenced using a hybrid approach; **bold** = type strain.

¥ Determined based on leafy gall and root inhibition assays described in a separate study (see associated text for reference).

§ D188 had 65,301,274 PE Illumina reads and 107,436 454 Jr. reads and A44a had 25,093,452 PE Illumina reads and 62,188,838 Illumina mate pair reads from a 3.0 kb library.

† Number greater than 1.0 kb in length.

### A pFiD188-like plasmid is present in most, but not all, phytopathogenic isolates of *Rhodococcus*

**Figure 3:**
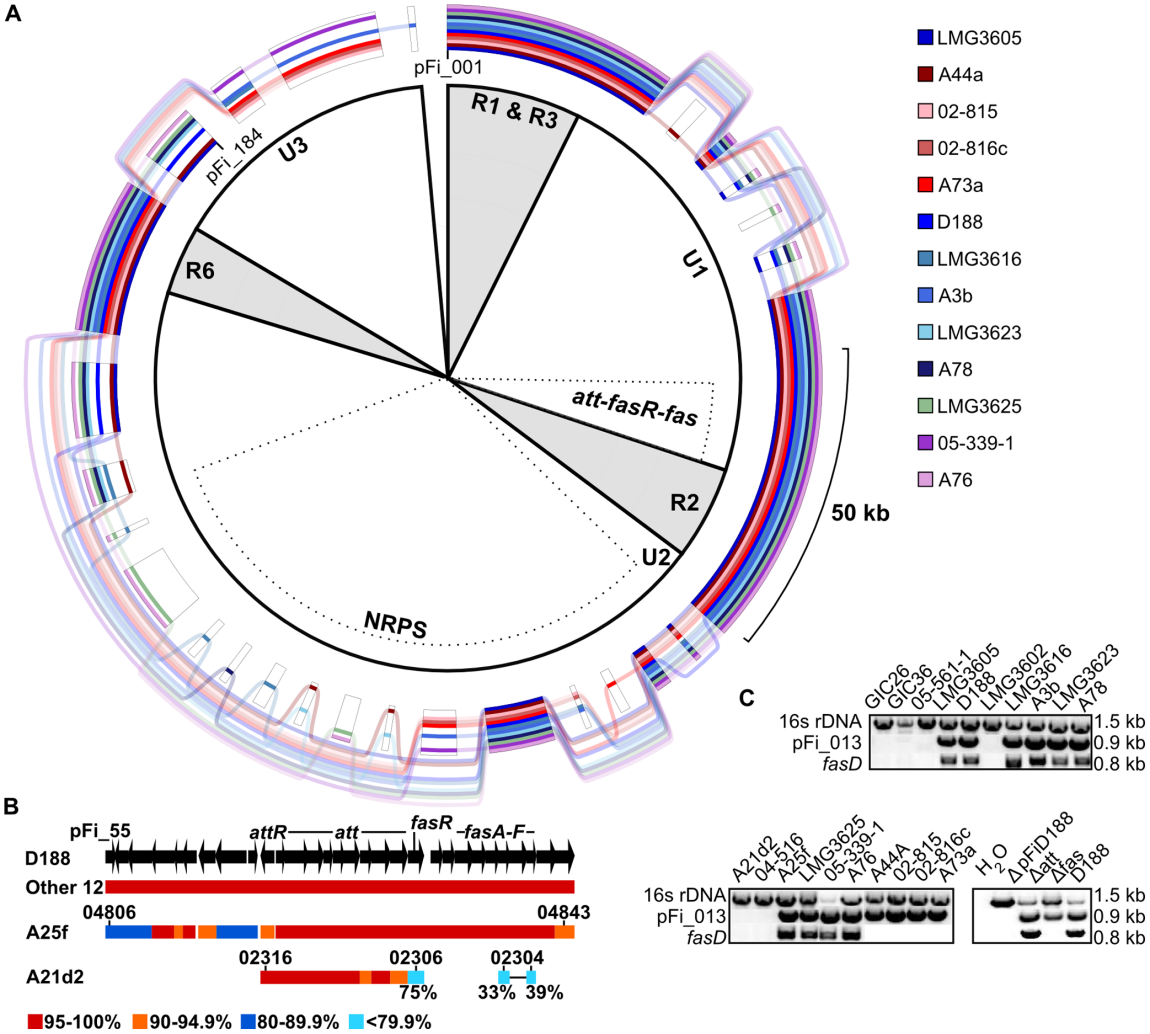
The *att*, *fasR*, and *fas* virulence loci are variable in organization among phytopathogenic isolates of *Rhodococcus*. (A) GenomeRing of the 13 pFiD188-like sequences. Colored lines, corresponding to each isolate, indicate presence of a block. Transparent lines skip over absent blocks and connect co-linear blocks. A minimum block size of 1 kb was used. The inner portion defines conserved (R) and unique (U) regions of pFiD188 from isolate D188, as previously reported. The *att*-*fas* virulence loci and the NRPS-encoding region are also indicated by dotted regions. (B) Homology and co-linearity of the pFi_55-pFi_84 regions in the 15 phytopathogenic isolates. Top: structure of the region of pFiD188 from pFi_55 to pFi_84, with arrows depicting CDSs and the direction indicating the strand in which they are encoded. For isolates with a linear plasmid (Other 12), A25f, and A21d2, bars indicate the presence and range of sequence similarity. Relevant locus ID numbers are shown without RF25f_ and RFA21d2_ tags. The two cyan bars connected by a single line represent a gene fusion. (C) PCR-based detection of virulence loci (*fasD*) and pFiD188-like (pFi-013) sequences. The 16s rDNA gene was used as a positive control. Products were resolved on a 1%, 1X TAE agarose gel. Estimated product sizes (kb) are listed along the side.

The linear plasmid, pFiD188 is necessary for the virulence of *R. fascians* D188 [14]. We therefore used the sequence of pFiD188 as a query to search the genome assemblies to examine its necessity for *Rhodococcus* virulence [19]. CDSs homologous to those along the entire length of pFiD188 are present in 13 of 15 genome sequences of phytopathogenic isolates (Fig. 3A). The sequences of *att*, *fasR*, and *fas* are conserved (85% of the 204 CDSs in this cluster of virulence CDSs are identical and the remaining 15% are >99% identical to corresponding loci of the pFiD188 sequence generated in this study; Fig. 3B). However, within these loci, we identified 24 sequence discrepancies that differed in comparison to the previously reported pFiD188 sequence [19]. Because all linear plasmid sequences from this study were identical at these positions, we concluded the published sequence had errors. Of the 18 affecting the translated sequences of CDSs, 17 resulted in single amino acid changes. One was an insertion of a guanine residue in *fasR*, relative to the published sequence, that leads to substantially longer translated sequences because of an upstream, in-frame ATG start codon.

A linear plasmid sequence is absent in two other pathogenic isolates, A25f and A21d2, and the five non-pathogenic isolates. PCR for a CDS located in the R1 regions of the pFiD188-like plasmids and implicated in their maintenance confirmed *in silico* predictions (Fig. 3C). Therefore, the linear plasmid is correlated with phytopathogenicity, but is not strictly necessary.

Between ∼40%–80% of the CDSs associated with the 13 linear plasmid sequences were identified as being acquired by HGT. Most are located in or at the border of the three “U” regions that were previously classified based on regions within pFiD188 of D188 that are unique in sequence relative to linear plasmids of other members of *Rhodococcus* [19]. Additionally, the gene gain/loss polymorphisms largely corresponded to the U regions and also correlated with evidence for HGT (Fig. 3A; Table S1). The pattern of insertions and deletions in the NRPS-encoding CDS of the U2 region is likely a reflection of limitations in assembling short reads exacerbated by the modularity of NRPS genes. The predicted functions for the polymorphic genes in the U regions did not provide strong evidence for a role in pathogen virulence (Table S1). Of the four conserved R1, R2, R3, and R6 regions, previously defined based on sequence similarities to other linear plasmids of *Rhodococcus*, there were fewer gene gain/loss polymorphisms but R3 and to some degree, R1 had evidence for HGT. In summary, results from the analysis of 13 linear plasmid sequences were consistent with previous reports in regards to the presence of conserved and unique regions, with higher incidences of recombination in the unique regions [19].

### The contribution of horizontal gene transfer to virulence evolution is limited in its scale

**Figure 4:**
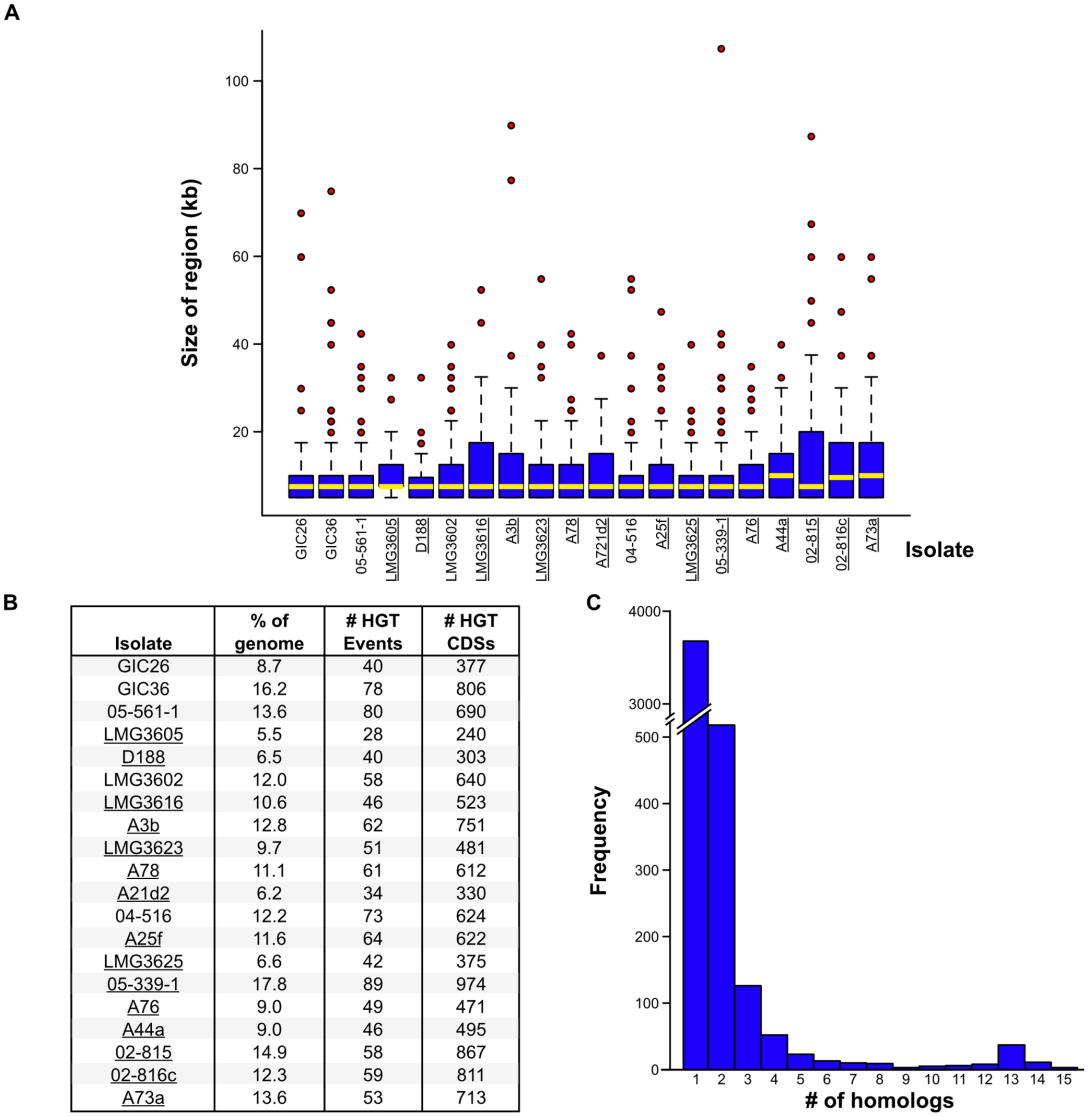
The scale of horizontal gene transfer varies among isolates. (A) Box-and-Whisker plots of regions acquired by HGT as a factor of size. Regions putatively acquired via HGT were identified using Alien Hunter, with minimum criteria of size ≥5 kb and minimum score ranging from 10.297 to 16.047. Bottom and top of the boxes indicate the first and third quartiles, respectively, with the yellow bar indicating the median. Whiskers delimit the lowest and highest data within 1.5 interquartile ranges of the lowest and highest quartiles, respectively. Red circles represent outliers. Pathogenic isolates are underlined. (B) Summary statistics of HGT for the sequenced isolates. Pathogenic isolates are underlined. (C) Histogram presenting the number of groups of CDSs as a factor of group size. All CDSs identified in regions putatively acquired by HGT were compared using BLAST analysis to determine groups of homologs. The numbers of groups of CDSs (y-axis) were enumerated and plotted according to the size of the groups (x-axis). Analysis was done for only the 15 phytopathogenic isolates.

Virulence genes of plant pathogens can be located in islands within the chromosome. We therefore searched the genomes for regions with signatures of HGT [26]. The average size for the identified regions was 11.5 kb (Fig. 4A). The scale of HGT was variable between isolates, with no clear correlation to phytopathogenicity (Fig. 4B). In phytopathogenic isolate LMG3605 for example, only 240 CDSs were identified within regions acquired by HGT and their total size represented just 5.5% of the genome. In contrast, non-pathogenic isolate GIC36 has 78 regions with ∼800 CDSs representing 16.2% of the total size of the genome as potentially acquired by HGT. Of all identified regions, some of the outliers were substantial in size, highlighted by the ∼107.5 kb region, encoding 103 CDSs, in 05-339-1 (Fig. 4A).

We compared the non-redundant set of 6,867 CDSs present in the horizontally acquired regions from all 20 isolates to a custom database consisting of CDSs gathered from non-phytopathogenic Actinomycetes. Approximately 33% of the translated *Rhodococcus* CDSs did not have a BLASTP hit and were thus considered specific to this group of *Rhodococcus* isolates. The only two significantly enriched clusters of orthologous genes (COGs) categories were “General functional prediction only” and “Function unknown”, as would be expected for a group of relatively poorly studied bacteria. Of the CDSs horizontally acquired by only the phytopathogenic isolates, the significantly enriched functional categories related to energy, metabolism, and general functions (Fig. S1A). A parallel analysis using Gene Ontology (GO) terms yielded similar results, with general functions in metabolism, transport, as well as transcription and translation being the more prominently identified terms (Fig. S1B). The few clusters that are likely associated with virulence are cytokinin and isoprenoid biosynthesis and metabolic processes.

Next, we reasoned that horizontally acquired candidate virulence genes should be conserved across the majority of the phytopathogenic isolates. An overwhelming majority of the CDSs associated with regions putatively acquired by HGT were present in only one or two of the phytopathogenic isolates (Fig. 4C). Even when we lowered our criteria and considered CDS that had homologs in approximately one-half of the phytopathogenic isolates, fewer than 100 homologous families were identified. Each of the families had homologs present on pFiD188. Overall, data suggest that other than the linear plasmid, HGT does not play a large-scale role in virulence evolution of phytopathogenic *Rhodococcus* and likely contributes more to the outside-host lifestyle of the bacteria.

### Few genes are conserved and unique to phytopathogenic isolates

**Figure 5:**
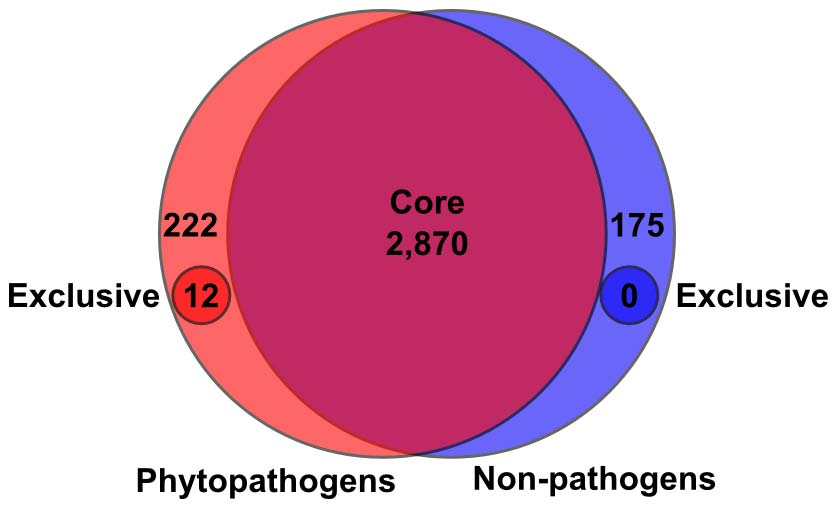
The pathogenic isolates have a small set of core coding sequences. A total of 16,733-redundant CDSs are present in the “pan” genome of the 24 isolates of *Rhodococcus* that were examined. Based on reciprocal best-hit BLASTP analysis of the translated sequences, 2,870 are core. Coding sequences core to pathogenic (red) and non-pathogenic (blue) genomes were similarly identified for the 15 and 9 isolates, respectively.

We hypothesized that, regardless of phylogenetic structure and HGT, the phytopathogenic isolates can be expected to have a core and exclusive set of CDS that distinguish them as pathogenic. We compared CDSs from 24 genome sequences, including the 20 from this study as well as *Rhodococcus* spp. AW25M09, JG-3, 29MFTsu3.1, and 114MTsu3.1 ([27]; GenBank BioProjects PRJNA200424, PRJNA195882, and PRJNA201196). The four additional isolates were identified from various environments, clustered with the phytopathogenic *Rhodococcus*, and lack loci known to be necessary for *Rhodococcus* phytopathogenicity (Creason and Chang, data not shown). Overall, we identified a “core” of 2,870 and a “pan” of 16,733 CDSs (Fig. 5). The COG categories in the “flexible” genome (13,863 CDSs) were enriched in categories related to energy and responding to the environment and its co-inhabitants (Fig. S2).

Because we were interested in identifying CDSs that are exclusive to pathogens, we compared pan and core genomes of the 15 phytopathogenic isolates to the pan and core genomes of the 9 non-pathogenic isolates (Fig. 5). The 15 pathogenic isolates have a core of 234 CDSs that were not also identified as core to the nine non-pathogenic isolates. However, only 12 of the 234 were classified as exclusive based on their complete absence from the nine non-pathogenic isolates; the other 222 CDSs are part of the flexible genome of the non-pathogenic isolates. The 12 exclusive CDSs correspond to the *att* locus (10 CDSs), *fasR*, and a putative FAD-binding monooxygenase-encoding gene (pFi_057 of pFiD188), all of which have members on pFiD188. The reciprocal best hit for the FAD-binding monooxygenase-encoding gene of A21d2 barely exceeded threshold, with an identity of only 35.2% suggesting it may not be a *bona fide* member of the gene family, as each isolate has large numbers of monooxygenase-encoding genes (Davis and Chang, data not shown). Most notably, the six *fas* CDSs were not identified as core to the pathogenic isolates because of the absence of *fas* from isolate A21d2. PCRs using oligonucleotides specific to each CDSs of the *fas* operon were negative (Figs. 3B and 3C; Table S1).

### The virulence loci of A25f and A21d2 are novel in structure and sequence

We used the pFiD188 sequence and reciprocal best-hit analysis to identify scaffolds of ∼190 kb and 18 kb in the A25f and A21d2 assemblies, respectively (Table S2). In A25f, 30 of the 33 best hit CDSs (RFA25f_04806-RFA25f_04843) are co-linear to their homologs on pFiD188, starting from nine CDSs upstream of the *att* locus and extending to two CDSs past the *fas* locus (Fig. 3B). The highest levels of sequence similarities were observed for *att* through *fas* (>95% identity; AttR = 94.5%). Despite that, the degree of sequence similarity was lower relative to the degree of similarity found among the 13 isolates that carry a linear plasmid like pFiD188. Analysis of flanking sequences did not provide any clues to the location of this scaffold in the genome because there were no regions of co-linearity with the D188 assembly. However, the scaffold lacked features typically associated with plasmids and the most parsimonious explanation is that a recombination event occurred between a portion of a pFiD188-like plasmid and the chromosome of A25f. Consistent with previously reported results, A25f delineates a small portion of the linear plasmid as necessary for phytopathogenicity [19].

In A21d2, only 11 of the 28 best hit CDSs in the 18 kb scaffold are co-linear to the U1 region, starting at RFA21d2_02316 (*attR*) and unexpectedly and abruptly ending after RFA21d2_02306, an AraC-like encoding CDS (*fasR*; Fig. 3B). We used thermal asymmetric interlaced (TAIL) PCR to determine flanking sequences. The resultant sequences were nearly 1 kb long, partially similar in sequence to each other, and mapped to separate single locations that coincided to a region between RFA21d2_03323 and RFA21d2_03324 within a 169 kb long scaffold, node_3. Consistent with previous searches, no *fas* operon was present in node_3 and no functional replacement of the Fas proteins could be predicted from the translated sequences of node_3. The A21d2 homolog of pFi_057 was not found in this block of co-linear CDSs and is instead located over 1 Mb away.

### The *fas* operon of A21d2 is replaced by a gene fusion encoding IPT and LOG domains

**Figure 6:**
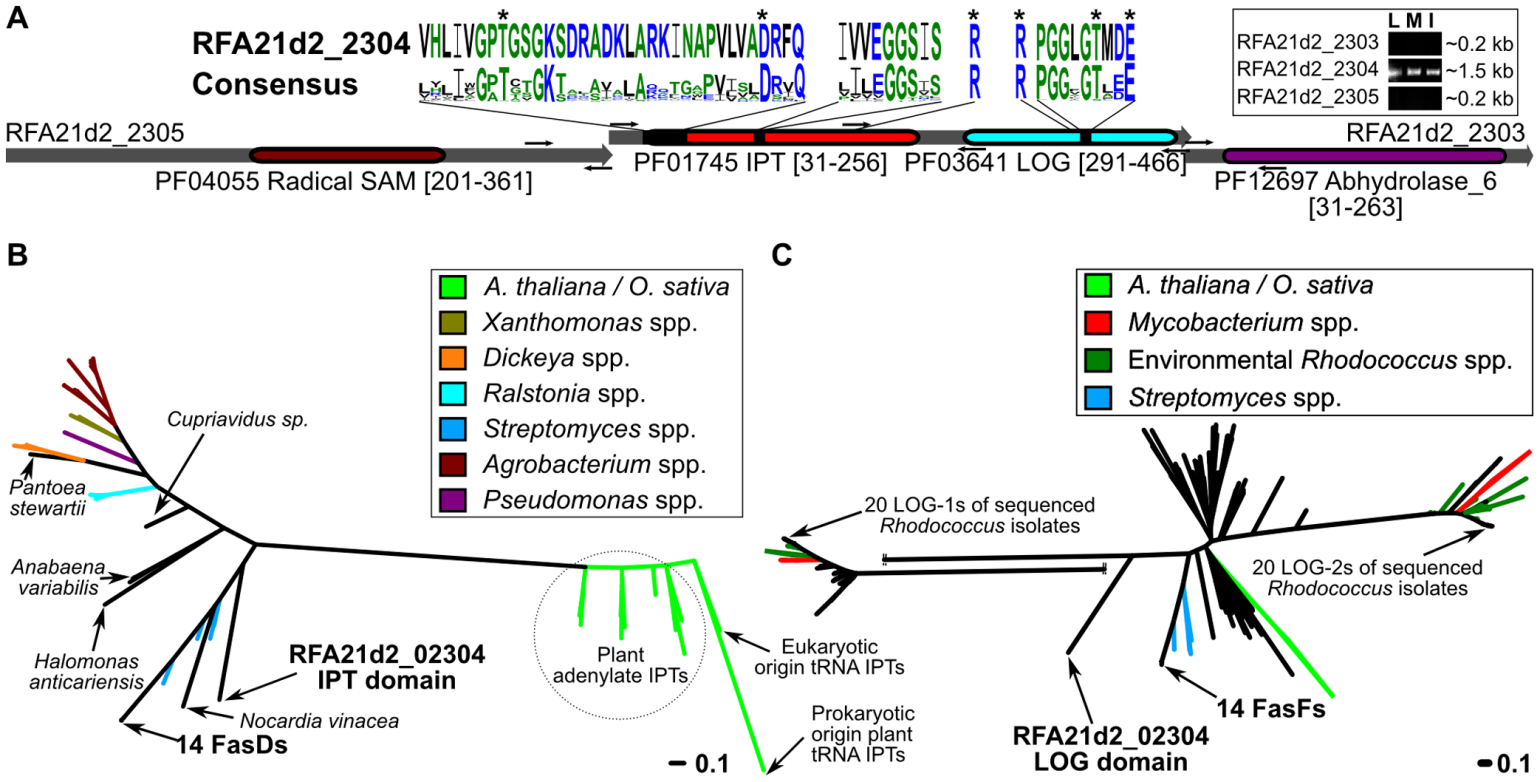
RFA21d2_02304 is a gene fusion unique to A21d2. (A) Structure and predicted functional domains for RFA21d2_02303-02305. Functional domains were identified using PFAM (PFxxxx) and mapped to each of the sequences. Sequence logos were generated and shown for the isopentyltransferase and phosphoribohydrolase domains. The best matching residues of RFA21d2_02304 are shown and mapped to their corresponding locations in the diagram. Amino acids are colored according to class. The “*” highlights conserved residues; the single “R” at positions 166 and 378 are not part of the sequence logos but are included to show their conservation. Small arrows show the locations of the binding sequences for oligonucleotides used in RT-PCR (see inset: L = LB; I = minimal media+leafy gall extracts; M = minimal media). RT-PCRs with oligonucleotides that span both overlapping regions were negative and are not shown. (B) Unrooted phylogenetic tree for IPTs. Scale bar = number of amino acid substitutions per site. (C) Unrooted phylogenetic tree for phosphoribohydrolases. Scale bar = number of amino acid substitutions per site. One branch was split for sizing purposes.

The absence of the *fas* operon from A21d2 was not expected. The locus is replaced by a novel cluster of three overlapping CDSs (Fig. 6A). The outer two CDSs, RFA21d2_02305 and _02303, are annotated as containing a single Radical SAM domain and an Abhydrolase_6 domain, respectively. Neither of the domains are present in the Fas proteins of pFiD188 and the single Radical SAM domain is different from those predicted for the translated sequences of *mtr1* and the homologous *mtr2*, both of which are located between *fasR* and the *fas* operon in pFiD188 [19]. RFA21d2_02305 and _02303 were not expressed in cells grown in any of the three media tested and not predicted to be involved in cytokinin metabolism (Fig. 6A).

Remarkably, RFA21d2_02304 is a chimera between *fasD-* and *fasF*-like sequences and the possibility of an assembly mistake was dismissed based on PCR and Sanger sequencing that confirmed the sequence of the locus (Fig. 6A; Table S2). Both IPT and cytokinin phosphoribohydrolase domains are present in RFA21d2_02304. Three residues necessary for IPT function and conserved in all IPT sequences previously examined by others are present in the N-terminal portion of RFA21d2_02304 [28]. There are also three conserved and predicted catalytic residues in the C-terminal portion of RFA21d2_02304 that are found in similar positions relative to other *bona fide* LOG proteins [29]. Unlike *fasD* of D188 and the CDSs flanking RFA21d2_02304, the chimera is expressed under what is considered non-inducing conditions (Fig. 6A; Fig. S3). These results indicated the three CDSs are expressed as independent monocistronic messages.

The two domains of RFA21d2_02304 formed branches distinct from the 14 other FasD and FasF homologs (Figs. 6B and 6C). The IPT region is more similar to FasD than the LOG region is to FasF, and as previously shown, IPTs from phytopathogenic *Rhodococcus* cluster separately from those of plants and other bacteria [21], [30]. The exceptions are IPTs of *Streptomyces* spp., which also encode the *fas* operon [31]. The LOG region of RFA21d2_02304 and FasF are more similar to LOG of plants than to either of the two homologs present in *Rhodococcus*. In all, the data suggested that RFA21d2_02304 was acquired horizontally from another source, as opposed to being derived from a series of recombination events within a pFiD188-like plasmid.

### Isopentenyladenine is the only cytokinin that specifically accumulates in extracts of D188 grown in culture and under inducing conditions

The absence of *fasA* from A21d2 is not consistent with the model that predicts the necessity for a mixture of cytokinin types (isopentenyladenine (iP), tZ, ciz-zeatin (cZ), and the methylthio derivatives of the latter two) for virulence [18], [20]. The 20 *Rhodococcus* isolates encode homologs of all the enzymes necessary for the methylerythritol phosphate (MEP) pathway, including a homolog of isopentenyl diphosphate (IPP) isomerase (IDI1) that catalyzes the isomerization of IPP to dimethylallyl diphosphate (DMADP; Fig. 1; Table S3). The MEP pathway synthesizes DMADP and the intermediate, (E)-4-hydroxy-3-methyl-but-2-enyl diphosphate (HMBDP), both of which are isoprenoid side chain donors that can be used by IPTs to synthesize the isopentenyladenine ribotide (iPAMP) and tZ ribotide (tZRMP; [32]–[35]). In contrast, homologs for most of the enzymes of the mevalonate (MVA) pathway that synthesizes DMADP were not identified (Table S3).

**Figure 7:**
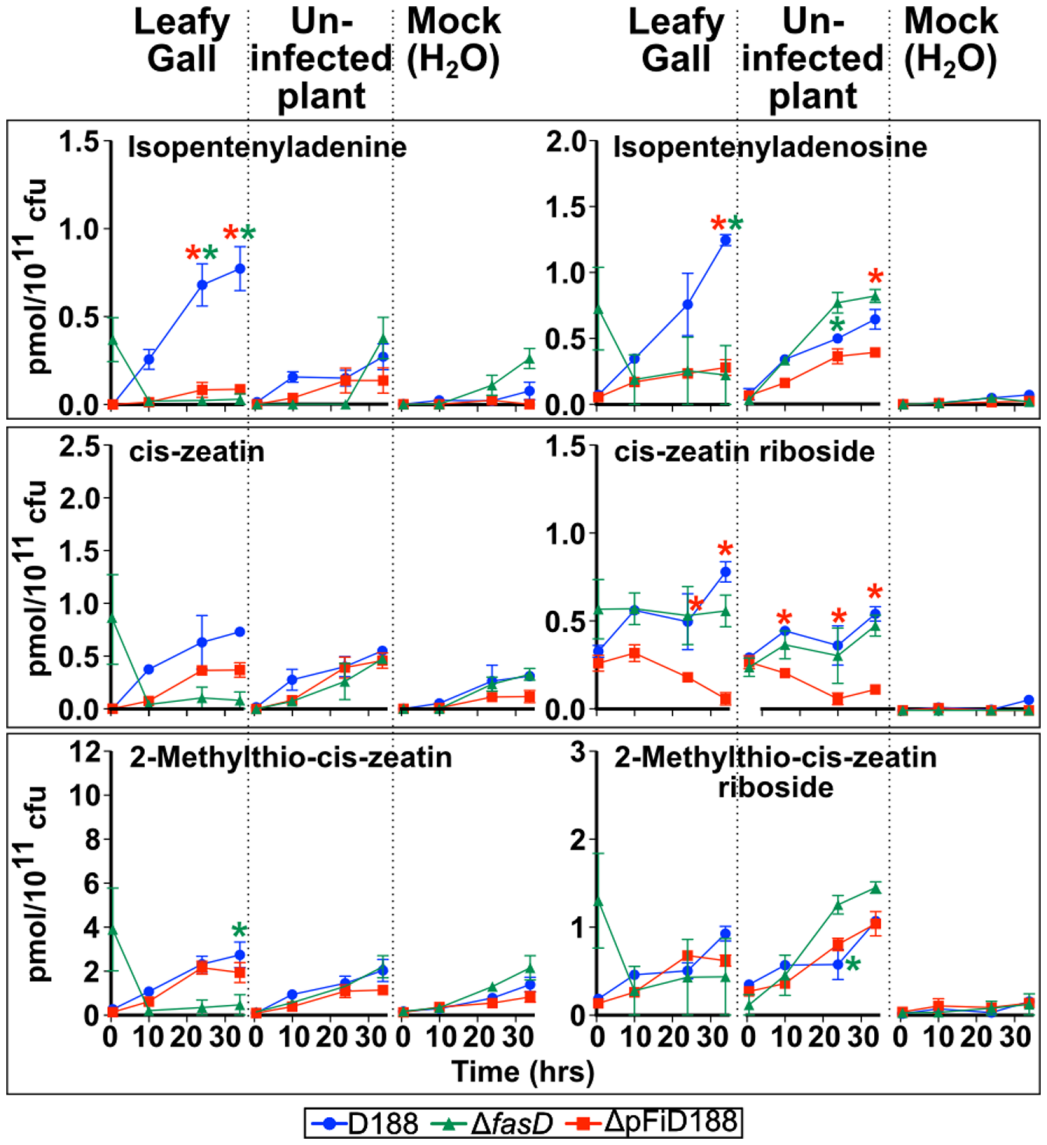
Isopentenyladenine is the only active cytokinin that accumulates in a *fasD* and leafy gall extract-dependent manner. Wild type D188 (blue), Δ*fasD* (green) and ΔpFiD188 (red), were grown in media augmented with extracts from leafy galls, uninfected plants, and water as a mock. Of 32 cytokinin types that were profiled, only eight were detected and the six most abundant types are shown. Change in concentration (y-axis) is presented for only the first four time points (x-axis). Colored “*” indicates significant difference in cytokinin accumulation in corresponding mutant genotype relative to wild type and corresponding time point (p-value threshold = 0.05). Experiments were repeated twice with similar results.

Because DMADP and HMBDP are potentially available, it is conceivable that the activities of FasD and FasF could enable the synthesis of both iP and tZ by *Rhodococcus* in culture. To test this, we profiled cytokinins from genetic variants of D188 grown in minimal media supplemented with extracts of leafy galls, uninfected plants, or H_2_O as a mock treatment. Of the 32 cytokinins that were profiled, only eight could be detected: iP, cZ, 2-methylthio-cis-zeatin (2MeScZ), their ribosides, as well as iPAMP and tZRMP; tZ was not detected (Table S4). iP was the only active cytokinin type that significantly accumulated specifically in preparations from bacteria grown in leafy gall extracts and in a *fasD/*pFiD188-dependent manner (Fig. 7). In contrast, none of the detectable Z-type cytokinins accumulated to significant levels in a treatment-dependent manner. All detectable cytokinin types started to accumulate at later time points in the Δ*fasD* mutant, independent of treatment (Fig. S4). Expression profiles of *attE* and *fasD* showed induced expression specific to bacterial cells grown in extracts of leafy galls, as previously reported (Fig. S2; [16]. Maximum expression was observed at 6 hours (10,000X and 100X more expression for *attE* and *fasD*, respectively, relative to expression in cells grown in extracts of uninfected plants) and rapidly declined to basal levels by 12 hours after growth. Data indicated that phytopathogenic *Rhodococcus* synthesizes only one active type of cytokinin in a *fasD*-and leafy gall-dependent manner.

### The *fasR* CDSs of A25f and A21d2 are substantially more polymorphic than other family members

**Figure 8:**
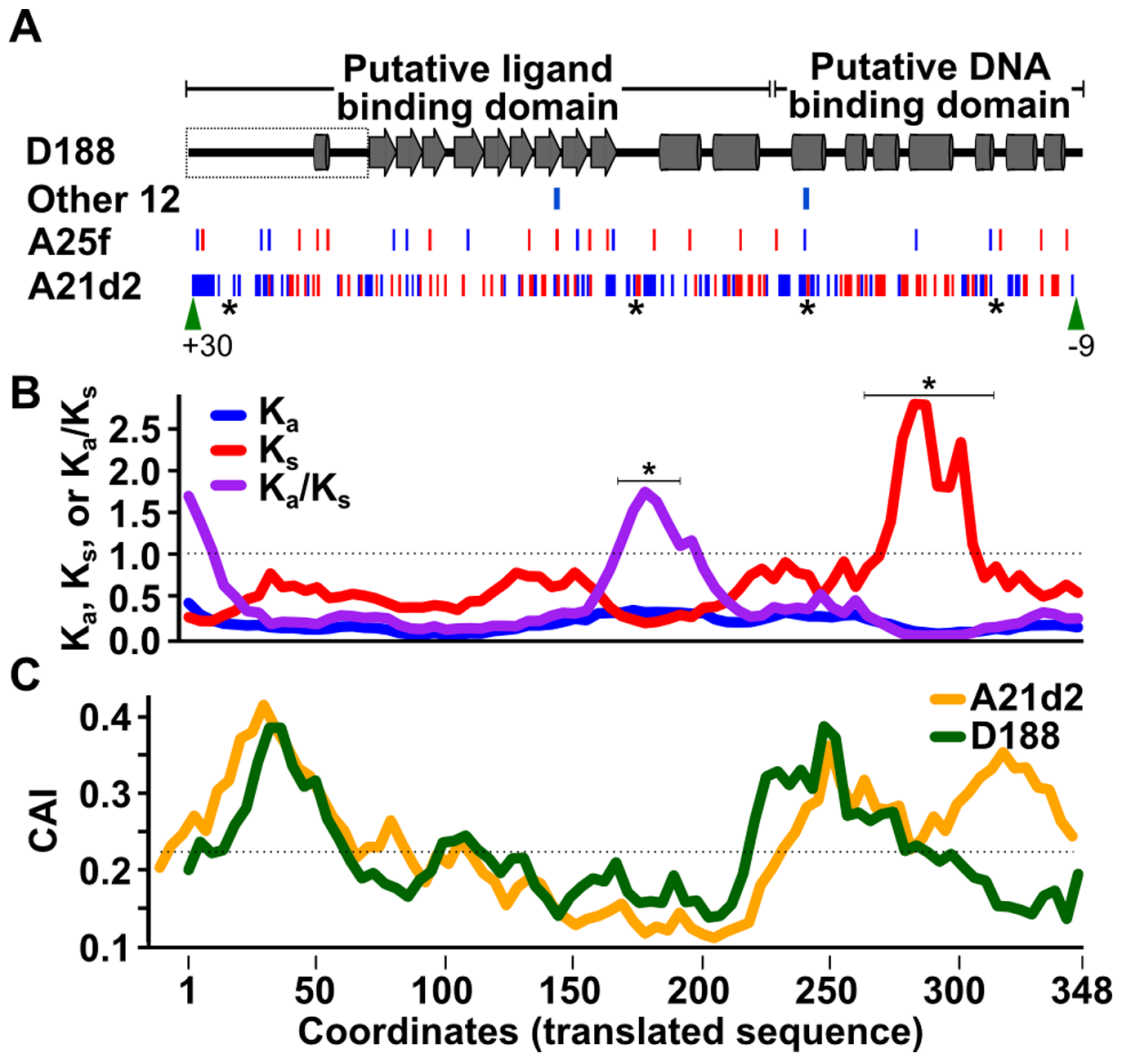
RFA25f_04833 and RFA21d2_02306 are polymorphic in sequence. (A) Predicted structure of the 348 amino acid FasR sequence. Functional domains are indicated along the top with arrows and barrels representing predicted β-sheets and α-helices, respectively. Boxed region = a region of *fasR* from D188 that is absent from the reported sequence. Synonymous (red) and non-synonymous substitutions (blue) and INDELs (green arrow heads with number of nucleotide differences indicated) are plotted according to their location in the sequences. Clusters of non-synonymous substitutions in *fasR* of A21d2 are denoted with, “*”. Substitutions for the other 12 FasR sequences (Other 12) represent the sum total of all sites with substitutions. (B) Sliding-window analysis of synonymous and non-synonymous substitutions in *fasR* of A21d2 paired with *fasR* of D188. (C) Sliding-window analysis of codon adaptation index in *fasR* of A21d2 and D188. The x-axis is based on coordinates of the translated sequence that are aligned to predicted structure shown in panel A.

Putative AraC-like encoding CDSs are located between the *att* operon and *fas* operon or *fas*-like CDSs in both A25f and A21d2 (RFA25f_04127 and RFA21d2_02306, respectively; Fig. 3). RFA25f_04127 and RFA21d2_02306 are predicted to be functional and clustered with *fasR* in a phylogenetic tree, supporting their membership to this family (bootstrap percentage of 100%; Fig. S5). However, the sequences of *fasR* are highly polymorphic (Fig. 8A). The translated sequence of the A25f CDS shares 97% identity with FasR of D188. In RFA21d2_02306, there are 343 SNPs (32% of all possible sites), of which 89, or 26% of the SNPs, are non-synonymous substitutions.

RFA21d2_02306 is nevertheless predicted to be under purifying selection (K_a_/K_s_ = 0.327; p-value = 9.3×10^−20^; paired with allele from D188). RFA21d2_02306 is also not optimized for translation (CAI = 0.221; 59 ribosomal protein encoding genes = 0.667; genome average = 0.530). This was not surprising since *fasR* includes an unusually high number of rare TTA leucine codons [16]. In attempts to model the patterns of substitutions, we mapped them onto the predicted secondary structure of FasR (Fig. 8A). Both domains of the predicted structure are similar to other AraC-like regulators [36]–[39]. One region within the putative ligand binding domain was identified as having a high K_a_/K_s_ and was significant in a permutation test (p-value = 0.033; Fig. 8B; [40]). The K_s_ values started trending upward thereafter within the putative DNA binding domain and culminated in a region with very high values (p-value = 0.04). This region overlaps two helix-turn-helix motifs predicted to be involved in binding DNA, based on alignments to AraC. In addition, a larger portion of this region correlated with above average CAI values in RFA21d2_02306 relative to FasR of D188 (Fig. 8C). Therefore, despite evidence for purifying selection and a high density of synonymous substitutions in the putative DNA binding domain, diversifying selection was identified in a putative ligand binding region.

## Discussion

Phytopathogenic *Rhodococcus* are unlike most plant pathogens. These bacteria are Gram-positive Actinobacteria with members capable of causing growth deformities and persisting, often through the life of the host plant. To gain insights into the evolution of *Rhodococcus* virulence and develop resources for understanding the mechanisms of phytopathogenesis, we determined and characterized the genome sequences for 20 isolates. Analyses suggested that the acquisition of just four functions is sufficient for diverse isolates of *Rhodococcus* to gain the trait of phytopathogenicity. Additionally, the discovery of a novel gene chimera and profiling of the exemplar isolate challenged the cytokinin mixture model of *Rhodococcus* virulence.

### *Rhodococcus* requires four horizontally acquired functions to be phytopathogenic

Virulence evolution of phytopathogenic *Rhodococcus* species can be modeled by the co-option mechanism proposed for *R. equi* [10], [24]. It has been previously reported that the chromosomal locus *vicA*, which encodes malate synthase that functions in the glyoxylate shunt pathway of the Krebs cycle, is necessary for full symptom development [41]. Additionally, the predicted dependency of the Fas proteins on the MEP pathway for the necessary substrate for cytokinin biosynthesis is another example of co-option (Fig. 1). Few other loci could be identified that had evidence for HGT and implicated in virulence. Only a miniscule fraction of the CDSs associated with regions with signatures of HGT were found conserved in pathogenic isolates and all of these CDSs have homologs encoded on a pFiD188-like plasmid (Fig. 4). Analysis of functional categories of CDSs associated with regions acquired via HGT also failed to support HGT as having a large-scale contribution in virulence evolution (Fig. S1). Similar findings were described for the COG categories enriched in the “flexible” genome (Fig. S2). The results are consistent with the linear plasmid being the key acquisition for virulence and the majority of horizontally acquired functions contributing to the adaptation of *Rhodococcus* to their outside-host environments. Indeed, *R. fascians* is considered a soil bacterium and closely related isolates have been isolated from extreme environments, suggesting an ability of these organisms to use a wide range of substrates [42]–[44].

### The structures and sequences of virulence loci of A25f and A21d2 are novel

We suggest that the horizontal acquisition of just four functions involved in secondary metabolism (*att*), gene transcription (*fasR*), cytokinin biosynthesis (*fasD*), and cytokinin activation (*fasF*) is sufficient for *Rhodococcus* to infect plants. Isolates A25f and A21d2 are unique among the sequenced samples. In A25f a recombination event likely occurred that introduced a block of CDSs from a linear plasmid into the chromosome. Gene fusions are generally predicted to occur by insertions and deletions between juxtaposed genes, such as those within an operon, and disseminated via HGT [45], [46]. However, for A21d2 a fusion would have had to occur between two CDSs separated by *fasE*. Stronger evidence against a direct fusion event comes from the observation that the IPT and LOG domains of RFA21d2_02304 formed branches clearly distinct from their homologs and that RFA21d2_02304 overlaps two CDSs absent from other sequenced isolates of *Rhodococcus* (Fig. 6). We therefore conclude that RFA21d2_02304 was acquired from another organism in what could be a non-orthologous displacement of the *fas* operon.

Integration of virulence loci into the chromosome is expected to confer a greater state of stability. However, the rarity of the virulence loci of A25f and A21d2 among the sequenced isolates is not consistent with this expectation. A potential explanation is that because the pFiD188-like linear plasmids are conjugative, the mobility of these vehicles may compensate for any lowered stability by enabling rapid dissemination throughout the population. This is highly plausible especially considering that acquisition of the four functions, most frequently vectored by a plasmid, is sufficient for genetically divergent *Rhodococcus* isolates to be phytopathogenic (Fig. 2). Alternatively, the events that led to the changes in A25f and A21d2 could have occurred relatively recently or the 20 isolates that were sequenced do not adequately reflect the diversity of the population. We also cannot ignore the likelihood for trade-offs that occur in non-obligatory pathogens that persist in both within and outside-host environments.

### The *att* locus is conserved in the phytopathogenic *Rhodococcus* isolates

The *att* locus is the most conserved in sequence and structure across all phytopathogenic isolates examined and consistently clustered with the *fas* locus (Fig. 3). Its product is also the only known target of host resistance [47]. Together, these data suggest the product of *att* is an important virulence determinant in *Rhodococcus* despite the attenuated phenotype of the Δ*att* mutant [17]. The infection methods used in the laboratory may possibly circumvent *att* dependence, since the Δ*att* mutant can cause disease if the host is wounded prior to infection. It has been suggested that *att* is important for the early transition of *R. fascians* from epiphyte to endophyte and its ability to ingress into host tissue [17]. Indeed, *attE* expression peaks to extremely high levels within six hours of growth in inducing media and rapidly decays thereafter (Fig. S3). Seemingly counter to the importance of *att* is its absence from *Streptomyces turgidiscabies*, which does have the *fas* operon and can form galls on plants ([31]; PRJNA185785). However, *att* may not be necessary because *S. turgidiscabies* does not ingress but rather, it primarily enters its host through wounds and natural openings.

### A new model for *Rhodococcus* virulence

The data generally supported the model identifying pFiD188 as necessary for *R. fascians* and the role of cytokinins in the pathology of plant-associated *Rhodococcus*. However, several lines of evidence presented in this study are consistent with a mechanism of virulence where only the iP cytokinin type, not a mixture of cytokinins, is synthesized and necessary for *Rhodococcus* phytopathogenicity (Figs. 1, 3, 6, and 7). We and others could not detect tZ and/or demonstrate its FasD-dependent synthesis in culture (Fig. 7; [18], [48]). The other two free base types that could be detected, cZ and 2MeScZ, accumulated independently of *fas* gene induction, similar to previous reports (Fig. 7; Fig. S4; [20], [21], [49]–[53]). The generation of tZ from cZ by the action of a cis-trans isomerase is not well substantiated, so it is unlikely that this could be a source of tZ [21].

Evidence also argues against FasD being able to directly produce significant levels of tZ even though bacterial IPTs can use HMBDP as a prenyl donor without the activity of a P450 monooxygenase [28], [32]. The Tzs IPT of *A. tumefaciens* has a hydrophilic region in the substrate-binding cavity that contributes to its ability to use both DMADP and HMBDP as substrates [28]. A substitution mutation that affected the hydrophilic region increased the specificity of *A. tumefaciens* Tzs for DMADP by ∼100-fold [28]. FasD of D188 has an alanine at a position that corresponds to one of the key hydrophilic residues of Tzs and the K_cat_/K_m_ value for FasD is 10X higher when DMADP is provided as a substrate, relative to HMBDP [18]. Therefore, while it is possible that FasD could use HMBDP to generate Tz, data suggests otherwise. Additionally, the novel gene chimera of A21d2 indicates that only the IPT and LOG functions are necessary for virulence (Figs. 3 and 6). The IPT domain of RFA21d2_02304 also has amino acid substitutions that affect the predicted hydrophilic region that contribute to the increased specificity of Tzs for HMBDP [28]. RFA21d2_02304 is thus not predicted to efficiently synthesize tZ.

The original model for *R. fascians* phytopathogenicity is predicated on the necessity of *fasA* for virulence [15]. However, the original *fasA* mutant was generated via insertion of a plasmid and the sufficiency of *fasA* in complementing the mutant was not tested, opening the possibility for polar effects. In addition, FasA has not been confirmed as having P450 monooxygenase activity and the absence of detectable levels of tZ indicate *fasA* may be dispensable. FasB and FasC, like deoxyxylulose 5-phosphate (DXP) synthase, the first enzyme of the MEP pathway, are members of thiamine pyrophosphate enzyme superfamilies [54]. We speculate that the possible redundancy in function could explain the dispensability of *fasB* and *fasC*. The superfluousness of *fasE* (cytokinin oxidase/dehydrogenase) for virulence solves the conundrum of why a pathogen has an operon that encodes an enzyme with a function that diametrically opposes those that synthesize and activate cytokinins as virulence factors. There are two explanations as to why functions predicted to be dispensable are still in the population of phytopathogenic *Rhodococcus* clades. The enzymatic functions of the FasA-C, and -E may be unnecessary but the proteins may be required for scaffolding and forming a protein complex for efficient cytokinin biosynthesis and activation. Alternatively or additionally, the CDSs may have not been purged because membership in the *fas* operon could confer a level of immunity to the effects of mutation or are important in contributing to the regulation of gene expression [55].

### New insights into cytokinin metabolism

The characterization of pathogen-encoded virulence proteins and molecules has provided many new insights into host growth, development, and signaling. Specifically, bacterial-encoded IPTs have contributed much to our understanding in cytokinin metabolism in plants [21]. The association of *fasF* with *fasD* in the *fas* operon was key to recognizing that LOG of plants is involved in cytokinin metabolism and provided evidence for the existence of the direct activation pathway of cytokinins [22]. The discovery of RFA21d2_02304 in this study raises the possibility that the phosphoribohydrolase complexes with IPT in other phytopathogenic *Rhodococcus* and also in plants. Gene fusions are important for increasing functional complexity through the generation of multi-domain proteins, often by uniting domains from those that physically interact and function in a common pathway. The low *in vitro* turnover rate of FasD led to the speculation of product inhibition [18]. The possibility of a complex with FasF indicates the potential for a direct activation of cytokinins and alleviation of inhibition. In A21d2, the fusion could be more efficient at coupling these two reactions, a possibility that will be addressed in future studies. It will also be interesting to determine if, and which, members of the IPT and LOG families form complexes in plants [56], [57].

## Materials and Methods

### Nucleic acid preparations, Sanger sequencing, and PCRs

Genomic DNA was extracted from cells grown directly from stocks. The Wizard Genomic DNA Purification Kit was used, according to the instructions of the manufacturer, to extract genomic DNA (Promega Corporation, Madison, WI, USA). Except for D188, isolates had undergone a limited number of passages.

Genomic DNA isolated from appropriate isolates was used as templates in PCRs with primers specific to the CDS (Table S5). Thermal Asymmetric Interlaced PCRs (TAIL) were done using nested oligonucleotides designed to Contig_21 and genomic DNA from A21d2, according to methods previously described (Table S5; [58]). Eight arbitrary degenerative primer pools were used in the amplification steps and successfully amplified products were sequenced and aligned to the A21d2 genome sequence.

For Sanger sequencing, products were treated with 2.5 U of Exonuclease I and 0.25 U of Shrimp Alkaline Phosphatase (SAP) for 40 minutes at 37°C and heat killed at 80°C for 20 minutes. Products were sequenced on an ABI 3730 DNA Analyzer in the Center for Genome Research and Biocomputing (CGRB) at Oregon State University.

Trizol Reagent (Life Technologies, Carlsbad, CA, USA) and ∼200 µl of 500 nm glass beads were added to *Rhodococcus* cells and disrupted using a FastPrep-24 homogenizer (MP Biomedical, Santa Ana, CA, USA) at 6.0 m/s for 1 minute. Samples were treated with DNase I (NEB, Ipswich, MA, USA), quantified and analyzed using a BioAnalyzer (Agilent Technologies, Santa Clara, CA, USA). First-strand cDNA was synthesized using 100 ng of total RNA and the Reverse Transcription System (Promega Corporation). One µl of the reverse transcription reaction was mixed with 10 µl LightCycler 480 SYBR Green I Master, 2.0 µl primer mix (250 nM each) and water for a final volume of 20 µl. Quantitative PCR was done on a LightCycler 480 System (Roche Applied Science, Germany). Gene expression was calculated using the deltadeltaCt method relative to the expression of the 16S gene and corresponding genes in the mock treatment [59]. For RT-PCR, first-strand cDNA was synthesized from 1.0 µg total RNA using Quantas (Life Technologies, Carlsbad, CA, USA). cDNA equivalent to 50 ng of starting RNA template was used in PCRs. Samples lacking reverse transcriptase were included as controls. Oligonucleotide sequences are available in Table S5.

### Next-generation sequencing, assembly, and annotation

Library construction and sequencing were done in the CGRB (Illumina GAIIx, HiSeq, and MiSeq, 454 Jr.), DNAVision SA (Illumina HiSeq of D188), or HTSF@UNC (Mate-pair sequencing of A44a).

Genomes were assembled using Velvet, with hash lengths of 75 [60]. Insert sizes were independently determined based on estimated fragment sizes from each library preparation. A44a was assembled using Velvet 0.7.55, D188 was assembled using Velvet 1.1.05, and the other 18 isolates were assembled using Velvet_1.2.08. For each genome, multiple assemblies were done, in which coverage cutoff, expected coverage, and hash length parameters were changed. The highest quality assembly was identified based on number of contigs and having a sum total size between 5–6 Mb. Contigs from isolate A44a were reordered with Mauve, using the genome sequence of *Rhodococcus jostii* RHA1 as a reference [61], [62]. All other assemblies were directly or indirectly re-ordered based on the genome sequence of A44a. Prokka was used to annotate genomes. Prokka identifies tRNAs, rRNAs, and CDSs using Aragaron, rnammer, and prodigal, respectively (*Prokka: Prokaryotic Genome Annotation System -* http://vicbioinformatics.com/). CDSs were annotated based on BLAST analysis to a database of genomes core to the *Rhodococcus* genus, including all whole-genome assemblies, followed by BLAST analysis to the UniProt database [63], [64]. The remaining CDSs were then used in searches against the HMM databases using HMMER3 [65].

### Phylogenetic analyses

Publically available sequences were gathered from the NCBI nr, nt, or wgs databases (http://www.ncbi.nlm.nih.gov/). Lists of sequences were manually curated. Sequences were aligned using L-INS-i algorithm in MAFFT and the most appropriate models of substitution were selected using the BIC selection method implemented in ProtTest 3 [66], [67]. Trees were generated using RAxML, with the -f a setting, and 1000 bootstrap replicates, unless otherwise noted [68]. Alignments were visualized and trimmed using Belvu [69]. Gblocks was used to trim the MLSA alignments with half gapped positions allowed [70]. Images were generated using the iTOL [71]. Only bootstrap values equal to or greater than 50 were shown.

For IPT-encoding genes, sequences were gathered by using RFD188_04926 (*fasD*) from D188 as the query in tBLASTn searches against both the nt and wgs NCBI databases. A total of 50 and 25 translated sequences, from the nt and wgs databases respectively, all IPT-encoding genes from *Arabidopsis thaliana* and *Oryza sativa,* and *fasD* from the *Rhodococcus* strains sequenced in this study were used to construct the tree. A similar approach was used for LOG-encoding genes with the exception that RFD188_04928 (*fasF*), RFD188_01289 (LOG-1), and RFD188_01290 (LOG-2) were used as query sequences. The top 50 hits for each query, 28 LOG family genes in the UniProtKB database and those found in the *Rhodococcus* strains sequenced in this study were used to construct the tree1. A total of 308 AraC-type sequences were identified, using BLASTP analysis, from the translated CDSs of all 20 *Rhodococcus* isolates (exceeded an e-value threshold of 1×10^−5^). Seventy of the sequences were used to construct the tree.

### Bioinformatic analyses

InterProScan was used to analyze the translated sequences of RFA21d2_02303-_2305 for domain analysis and generation of sequence logos [72]. Sequences from IPR005269 (LOG family, 9681 sequences) and IPR002648 (IPT domain, 144 sequences) were downloaded from the InterPro database and aligned using MAFFT–auto and L-INS-i, respectively [67], [73]. Sequences without the conserved domains were removed, leaving 9648 and 75 sequences, respectively. Logos were generated using WebLogo 3 (http://weblogo.threeplusone.com/; [74]).

For reciprocal best-hit BLAST analysis, the translated sequences of all CDSs of pFiD188 were used as queries in BLASTP searches of the A25f and A21d2 assemblies. We used the pFiD188 sequence from D188 generated in this study because of the discrepancies between it and the deposited sequence (JN093097; [19]). Top hitting homologs (p-value threshold = 1×10^−5^) were used as queries in reciprocal BLASTP analysis of the D188 assembly. Results were confirmed with tBLASTn searches.

The core and flexible genomes were identified using reciprocal best-hit BLASTP analysis of translated CDSs. Translated sequences were iteratively added into groups of orthologs, starting with strain D188. Threshold cut-offs of 35% identity and at least 50% gene coverage of the subject by the query were used.

Secondary structure predictions were made using jpred and visualized using jalview [75], [76]. ParaAT was used to generate alignments as input for Ka/Ks calculator [77], [78]. A 120-nucleotide window was slid along the sequence in 12 nucleotide increments for analysis of Ka/Ks and codon usage. For the permutation test, the ParaAT-aligned sequences were split based on aligned codons and the order of the codons was randomly permuted 1000 times. Values were calculated for each of the permuted sequences, using the same sliding window approach. We enumerated the number of times four (Ka and Ka/Ks) and six (Ks) consecutive windows were found in the permutations. Threshold values for window peaks were set at 1.5 * (standard deviation) from the mean for Ka and 2 * (standard deviation) from the mean for Ks and Ka/Ks, in relation to the initial aligned *fasR* sequences.

The SuperGenome based on the 13 pFiD188-like sequences was aligned with Mauve then generated and visualized using GenomeRing [62], [79].

Alien hunter (default settings) was used to predict regions acquired by horizontal gene transfer [26]. A custom BLAST database was generated using the translated CDSs from *Streptomyces coelicolor* A3, *Nocardia farcinica*, *Corynebacterium diphtheriae* NCTC 13129, *Gordonia* KTR9, *Mycobacterium tuberculosis* H37Rv, *Rhodococcus jostii* RHA1, *Rhodococcus opacus* B4, *Rhodococcus equi* 103S, and *Rhodococcus erythropolis* PR4 [24], [61], [80]–[86]. Homology was determined based on BLASTP top-hit analysis (threshold cut-offs = >50% in length and ≥35% identity).

WebMGA (default settings) and Revigo (Allowed similarity = 0.9) were used to assign genes to Cluster of Orthologous Groups and Gene Ontology (GO) categories, respectively [87], [88]. Revigo was also used to analyze GO categories. Fisher’s exact test was used to determine statistically significant differences in COG assignments (p-value≤0.01).

Graphs were generated in R [89].

Inkscape was used to design line drawings and figures (inkscape.org/).

### Bacterial strains and growth conditions

The 20 isolates of *Rhodococcus* (Table 1) were grown in Luria-Bertani (LB) media at 28°C. When appropriate, media were amended with 30 µg/ml kanamycin.

### Plant growth conditions and plant infection

For root inhibition assays, *N. benthamiana* was germinated on MS agar plates (half-strength MS, 0.5 M MES). Three-day-old germinated seedlings were inoculated with suspensions of *Rhodococcus* (OD_600_ = 0.5; 10 mM MgCl_2_ buffer) or mock inoculated (buffer only) and grown vertically for 1 week at room temperature with 16 hours of light. Plantlets were photographed and ImageJ was used to measure root lengths [90]. Comparisons for each *Rhodococcus* isolate were made relative to average root length of corresponding seedlings inoculated with ΔpFiD188 in the same experimental replicate. Tukey’s HSD test was used to determine significance.

Leafy galls were induced in 3–4-week-old *N. benthamiana* plants, as previously described [14]. The apical meristems of plants were pinched using forceps and inoculated with 10 µl of *Rhodococcus* (OD_600_ = 0.5; 10 mM MgCl_2_ buffer). Plants were grown under conditions described above and assessed weekly. Leafy galls were photographed at 4 weeks post-inoculation. For leafy gall extracts, 4 week-old leafy galls were excised from plants, ground in sterile water using a mortar and pestle, and filter sterilized (22 µm filter). Extracts were frozen at −80°C until use. Induction was done as previously described [91].

### Cytokinin profiling

Cytokinins were extracted from *R. fascians* D188 and its variants and quantified, using previously published methods [91], [92]. All supernatants were spiked with deutered cytokinin standards, including [^2^H_6_]iP, [^2^H_6_][9R]iP, [^2^H_6_](9G)iP, [^2^H_6_](7G)iP, [^2^H_5_]DHZ, [^2^H_5_][9R]DHZ, [^2^H_5_](9G)DHZ, [^2^H_5_](7G)DHZ, [^2^H_5_](OG)DHZ, [^2^H_5_](OG)[9R]DHZ, [^2^H_5_]2MeScZ, [^2^H_5_]2MeScZR, [^2^H_6_]MS-iP and [^2^H_6_]MS-iPA (Table S5; Olchemlm Ltd., Olomouc, Czech Republic). The pH of the samples was adjusted to pH 7 and samples were purified on a DEAE-Sephadex column (2 ml, HCO_3_^−^) with a RP-C18 column coupled underneath. After washing with ddH_2_O, the fraction containing the cytokinin free bases, ribosides, and glucosides was eluted with 80% methanol. Eluates were dried under vacuum, diluted with PBS and immunoaffinity purified using isoprenoid cytokinin IAC columns (Olchemlm Ltd.). After elution with ice-cold 100% methanol, eluates were dried in vacuum and stored at −20°C until further analysis. Cytokinin-phosphates (retained in the DEAE column) were eluted with NH_4_HCO_3_ and concentrated on a RP-C18 column before elution with 80% methanol. After vacuum concentration and dissolution with 0.01M Tris-HCl (pH 9.6), the cytokinin-phosphates fraction was treated with alkaline phosphatase and further immunoaffinity purified as described above.

Cytokinins were quantified using an electrospray ACQUITY TQP UPLC-MS/MS device (Waters, Micromass Ltd., United Kingdom). Samples (10 µl) were injected into an ACQUITY TQP UPLC BEH C18 column (1.7 µm×2.1 mm×50 mm, Waters) and eluted with ammonium acetate (1.0 mM) in 10% methanol (A) and 100% methanol (B). The UPLC gradient was as follows: linear gradient of 100% A to 55.6% A and 44.4% B in 8 minutes, followed by a wash with 100% B and an equilibration to initial conditions with 100% A at a flow rate of 0.3 ml/min. The effluent was introduced into the electrospray source at a temperature of 150°C. Quantitative analysis was carried out using the internal standard ratio methods and the deuterated isotopes. The ESI(+)-MRM (multiple reactant monitoring) mode was used for quantification based on specific diagnostic transitions for the different compounds analyzed (Table S6). Chromatograms were processed using the Masslynx software (Waters) and the concentrations were calculated following the principles of isotope dilution.

Two-way analysis of variance followed by Dunnett’s multiple comparison test were used to determine statistical significance. The two-way analysis of variance examined the influence of both time and bacterial strain on cytokinin levels assuming equal variance across both groups. Dunnett's test was used to compare cytokinin levels in Δ*fasD* and ΔpFiD188 to D188, within each time point, assuming equal variance among the treatment strains.

## Supporting Information

Figure S1 **Analysis of functional categories associated with coding sequences in regions acquired via HGT** (A) Analysis of clusters of orthologous groups (COGs). COGs of CDSs exclusive to pathogenic isolates were compared to those common among all 20 isolates. CDSs putatively acquired by HGT were categorized as exclusive to two or more pathogenic isolates (dark red) and those present in two or more isolates regardless of phytopathogenicity trait (blue). CDSs were assigned to COG functional groups (x-axis) and displayed as the percent of COGs per category relative to the total number of COGs assigned (y-axis). (B) Scatterplot of GO functional categories. GO terms were assigned to all coding sequences identified in pathogenic isolates and in regions potentially acquired via HGT. The semantic similarities were calculated, GO terms representative of clusters were generated, and plotted in a scatterplot. Clusters (circles) are placed based on semantically similarities, with more similar clusters placed closer to each other. Plot sizes represent the frequency of GO terms of a cluster relative to the total number of terms in the GO database. Heatmap represents semantic uniqueness, calculated relative to all terms in the list. High-level functions are indicated for clusters of the more frequently observed terms.
(EPS)

Figure S2 **Analysis of functional categories associated with the core and flexible genomes** COGs of CDSs that were core (blue) to all 24 isolates were compared to those in the flexible (red) genome. CDSs were assigned to COG functional groups (x-axis) and displayed as the percent of COGs per category relative to the total number of COGs assigned (y-axis).
(EPS)

Figure S3 **Marker genes of the** ***att*** **and** ***fas*** **operon were induced in the presence of leafy gall extract.** D188 was grown in media augmented with extracts from leafy galls (red), uninfected plants (blue), or water (mock). Samples were taken and RNA was extracted at the times indicated. Expression of *attE* and *fasD* was determined using qRT-PCR and calculated as expression relative to 16S and corresponding genes in D188 grown in a mock-treated culture.
(EPS)

Figure S4 **Complete cytokinin profiling data.** Three genotypes of D188, wild type (blue), Δ*fasD* (green) and ΔpFiD188 (red), were grown in media augmented with extracts from leaf galls, uninfected plants, and water as a mock. Of 32 cytokinin types that were profiled, only eight were detected and the six most abundant types are shown (iP: isopentenyladenine; cZ: cis-zeatin; 2MeScZ: 2-methylthio-cis-zeatin; and their ribosides). Change in concentration (y-axis) is presented for all time points (x-axis). Colored “*” indicates significant difference in cytokinin accumulation in corresponding mutant genotype relative to wild type and corresponding time point (p-value threshold = 0.05). Experiments were repeated twice with similar results.
(EPS)

Figure S5 **RFA25f_04833 and RFA21d2_02306 are members of the** ***fasR*** **family.** The alignment of the translated sequences for predicted AraC-type transcriptional regulators was used to generate a phylogenetic tree. Only candidates from the original 20 isolates were included. A single candidate AraC-type transcriptional regulator from *Rhodococcus jostii* RHA1 was used as the root. Genes are labeled by locus tag or with GI number (*R. jostii* RHA1). The FasR sequences are in bold. Node values indicate bootstrap percentages out of 1000 iterations. The scale bar represents the mean number of amino acid substitutions per site.
(EPS)

Table S1 **Predicted CDSs of the thirteen pFiD188-like sequences.** *Regions were designated based on previously published determinations; ^§^Functions were based on the annotations of the query sequences described in this study. The query sequences were derived from the CDS of the first isolate in which it appears in the table. Homologs were identified using reciprocal best BLAST hit analysis.
(XLSX)

Table S2 **Reciprocal best BLAST hit analysis of pFiD188 against the genome assemblies of A25f and A21d2.** *Locus identifiers from previously published pFID188 linear plasmid sequence; ^§^Based on this study.
(PDF)

Table S3 **Homologs of enzymes of the methylerythritol phosphate** **pathway are present in all 20 isolates of** ***Rhodococcus*** **.** *Locus; % identity; BLAST e-value. Column abbreviations: Deoxyxylulose 5-phosphate synthase (DXPS); DXP reductiosomerase (DXPR); isopentenyl diphosphate isomerase (IDI1); 4-diphosphocytidyl-2C-methyl-D-erythirol synthase (CMS); 4-(cytidine 5′-diphospho)-2-C-methyl-D-erythritol kinase (CMK); 2-C-methyl-D-erythritol 2,4-cyclodiphosphate synthase (MCS); hydroxy-2-methyl-2-(E)-butenyl 4-diphosphate synthase (HDS); hydroxy-2-methyl-2-(E)-butenyl 4-diphosphate reductase (HDR); No homologs of the MEP pathway were identified: 3-hydroxy-3-methylglutaryl-CoA synthase (HMGS; *N. farcinia*; YP_118423); 3-hydroxy-3-methylglutaryl-CoA reductase (HMGR; class I; *N. farcinia*; YP_118422); Mevalonate Kinase (MVK; *N. farcinia*; YP_118418); Phosphomevalonate kinase (PMK; *N. farcinia*; YP_118420); Mevalonate-5-decarboxylase (MDC; *N. farcinia*; YP_118419). YP_118418 identified homologs in isolates of sub-clades i and ii but e-values ranged from 6e-10 ∼ 3e-10 and CDSs are annotated as galactokinase. ^∧^Genbank ID number of sequence used for BLAST searches.
(PDF)

Table S4 **Cytokinin types that were profiled from D188.**
(PDF)

Table S5 **Sequences of oligonucleotides used in this study.**
(PDF)

Table S6 **Parent ions and diagnostic transitions used in multiple reactant monitoring (MRM) for the analysis of cytokinins by ACQUITY TQP UPLC-MS/MS.**
(PDF)

## References

[1] Win J, Chaparro-Garcia A, Belhaj K, Saunders DGO, Yoshida K, et al (2012) Effector biology of plant-associated organisms: concepts and perspectives. Cold Spring Harb Symp Quant Biol 77: 235–247 10.1101/sqb.2012.77.015933 23223409

[2] Xin X-F, He SY (2013) *Pseudomonas syringae* pv. tomato DC3000: a model pathogen for probing disease susceptibility and hormone signaling in plants. Annu Rev Phytopathol 51: 473–498 10.1146/annurev-phyto-082712-102321 23725467

[3] Brown JKM, Tellier A (2011) Plant-parasite coevolution: bridging the gap between genetics and ecology. Annu Rev Phytopathol 49: 345–367 10.1146/annurev-phyto-072910-095301 21513455

[4] Juhas M, van der MeerJR, Gaillard M, Harding RM, Hood DW, et al (2009) Genomic islands: tools of bacterial horizontal gene transfer and evolution. FEMS Microbiol Rev 33: 376–393 10.1111/j.1574-6976.2008.00136.x 19178566 PMC2704930

[5] Day WA, Fernández RE, Maurelli AT (2001) Pathoadaptive mutations that enhance virulence: genetic organization of the *cadA* regions of *Shigella* spp. Infect Immun 69: 7471–7480 10.1128/IAI.69.12.7471-7480.2001 11705922 PMC98836

[6] Ma W, Dong FFT, Stavrinides J, Guttman DS (2006) Type III effector diversification via both pathoadaptation and horizontal transfer in response to a coevolutionary arms race. PLoS Genet 2: e209 10.1371/journal.pgen.0020209 17194219 PMC1713259

[7] Gürtler V, Mayall BC, Seviour R (2004) Can whole genome analysis refine the taxonomy of the genus *Rhodococcus*? FEMS Microbiol Rev 28: 377–403. 15449609 10.1016/j.femsre.2004.01.001

[8] Larkin MJ, Kulakov LA, Allen CCR (2005) Biodegradation and *Rhodococcus*–masters of catabolic versatility. Curr Opin Biotechnol 16: 282–290 10.1016/j.copbio.2005.04.007 15961029

[9] Francis I, Holsters M, Vereecke D (2010) The Gram-positive side of plant-microbe interactions. Environ Microbiol 12: 1–12 10.1111/j.1462-2920.2009.01989.x 19624707

[10] Stes E, Vandeputte OM, Jaziri El M, Holsters M, Vereecke D (2011) A successful bacterial coup d’état: how *Rhodococcus fascians* redirects plant development. Annu Rev Phytopathol 49: 69–86 10.1146/annurev-phyto-072910-095217 21495844

[11] Cornelis K, Ritsema T, Nijsse J, Holsters M, Goethals K, et al (2001) The plant pathogen *Rhodococcus fascians* colonizes the exterior and interior of the aerial parts of plants. Molecular Plant-Microbe Interactions 14: 599–608 10.1094/MPMI.2001.14.5.599 11332724

[12] Putnam ML, Miller ML (2007) *Rhodococcus fascians* in herbaceous perennials. Plant Dis 91: 1064–1076 10.1094/PDIS-91-9-1064 30780643

[13] Vereecke D, Burssens S, Simón-Mateo C, Inzé D, Van Montagu M, et al (2000) The *Rhodococcus fascians*-plant interaction: morphological traits and biotechnological applications. Planta 210: 241–251. 10664130 10.1007/PL00008131

[14] Crespi M, Messens E, Caplan AB, Van Montagu M, Desomer J (1992) Fasciation induction by the phytopathogen *Rhodococcus fascians* depends upon a linear plasmid encoding a cytokinin synthase gene. EMBO J 11: 795–804. 1547783 10.1002/j.1460-2075.1992.tb05116.x PMC556518

[15] Crespi M, Vereecke D, Temmerman W, Van Montagu M, Desomer J (1994) The *fas* operon of *Rhodococcus fascians* encodes new genes required for efficient fasciation of host plants. J Bacteriol 176: 2492–2501. 8169198 10.1128/jb.176.9.2492-2501.1994 PMC205384

[16] Temmerman W, Vereecke D, Dreesen R, Van Montagu M, Holsters M, et al (2000) Leafy gall formation is controlled by *fasR*, an AraC-type regulatory gene in *Rhodococcus fascians* . J Bacteriol 182: 5832–5840. 11004184 10.1128/jb.182.20.5832-5840.2000 PMC94707

[17] Maes T, Vereecke D, Ritsema T, Cornelis K, Thu HN, et al (2001) The *att* locus of *Rhodococcus fascians* strain D188 is essential for full virulence on tobacco through the production of an autoregulatory compound. Mol Microbiol 42: 13–28. 11679063 10.1046/j.1365-2958.2001.02615.x

[18] Pertry I, Václavíková K, Gemrotová M, Spíchal L, Galuszka P, et al (2010) *Rhodococcus fascians* impacts plant development through the dynamic *fas*-mediated production of a cytokinin mix. Molecular Plant-Microbe Interactions 23: 1164–1174 10.1094/MPMI-23-9-1164 20687806

[19] Francis I, De Keyser A, De Backer P, Simón-Mateo C, Kalkus J, et al (2012) pFiD188, the Linear Virulence Plasmid of *Rhodococcus fascians* D188. Molecular Plant-Microbe Interactions 25: 637–647 10.1094/MPMI-08-11-0215 22482837

[20] Pertry I, Václavíková K, Depuydt S, Galuszka P, Spíchal L, et al (2009) Identification of *Rhodococcus fascians* cytokinins and their modus operandi to reshape the plant. Proc Natl Acad Sci USA 106: 929–934 10.1073/pnas.0811683106 19129491 PMC2630087

[21] Frébort I, Kowalska M, Hluska T, Frébortová J, Galuszka P (2011) Evolution of cytokinin biosynthesis and degradation. J Exp Bot 62: 2431–2452 10.1093/jxb/err004 21321050

[22] Kurakawa T, Ueda N, Maekawa M, Kobayashi K, Kojima M, et al (2007) Direct control of shoot meristem activity by a cytokinin-activating enzyme. Nature 445: 652–655 10.1038/nature05504 17287810

[23] Takei K, Yamaya T, Sakakibara H (2004) Arabidopsis CYP735A1 and CYP735A2 encode cytokinin hydroxylases that catalyze the biosynthesis of trans-Zeatin. J Biol Chem 279: 41866–41872 10.1074/jbc.M406337200 15280363

[24] Letek M, González P, Macarthur I, Rodríguez H, Freeman TC, et al (2010) The genome of a pathogenic *Rhodococcus*: cooptive virulence underpinned by key gene acquisitions. PLoS Genet 6: e1001145 10.1371/journal.pgen.1001145 20941392 PMC2947987

[25] Bargen von K, Haas A (2009) Molecular and infection biology of the horse pathogen *Rhodococcus equi* . FEMS Microbiol Rev 33: 870–891 10.1111/j.1574-6976.2009.00181.x 19453748

[26] Vernikos GS, Parkhill J (2006) Interpolated variable order motifs for identification of horizontally acquired DNA: revisiting the *Salmonella* pathogenicity islands. Bioinformatics (Oxford, England) 22: 2196–2203 10.1093/bioinformatics/btl369 16837528

[27] Hjerde E, Pierechod MM, Williamson AK, Bjerga GEK, Willassen NP, et al (2013) Draft Genome Sequence of the Actinomycete *Rhodococcus* sp. Strain AW25M09, Isolated from the Hadsel Fjord, Northern Norway. Genome Announc 1: e0005513 10.1128/genomeA.00055-13 23516194 PMC3593323

[28] Sugawara H, Ueda N, Kojima M, Makita N, Yamaya T, et al (2008) Structural insight into the reaction mechanism and evolution of cytokinin biosynthesis. Proc Natl Acad Sci USA 105: 2734–2739 10.1073/pnas.0707374105 18258747 PMC2268205

[29] Jeon WB, Allard STM, Bingman CA, Bitto E, Han BW, et al (2006) X-ray crystal structures of the conserved hypothetical proteins from *Arabidopsis thaliana* gene loci At5g11950 and AT2g37210. Proteins 65: 1051–1054 10.1002/prot.21166 17048257

[30] Kakimoto T (2003) Biosynthesis of cytokinins. J Plant Res 116: 233–239 10.1007/s10265-003-0095-5 12721785

[31] Joshi MV, Loria R (2007) *Streptomyces turgidiscabies* possesses a functional cytokinin biosynthetic pathway and produces leafy galls. Molecular Plant-Microbe Interactions 20: 751–758 10.1094/MPMI-20-7-0751 17601163

[32] Krall L, Raschke M, Zenk MH, Baron C (2002) The Tzs protein from *Agrobacterium tumefaciens* C58 produces zeatin riboside 5′-phosphate from 4-hydroxy-3-methyl-2-(E)-butenyl diphosphate and AMP. FEBS letters 527: 315–318. 12220681 10.1016/s0014-5793(02)03258-1

[33] Rodríguez-Concepción M, Boronat A (2002) Elucidation of the methylerythritol phosphate pathway for isoprenoid biosynthesis in bacteria and plastids. A metabolic milestone achieved through genomics. Plant Physiology 130: 1079–1089 10.1104/pp.007138 12427975 PMC1540259

[34] Sakakibara H, Kasahara H, Ueda N, Kojima M, Takei K, et al (2005) *Agrobacterium tumefaciens* increases cytokinin production in plastids by modifying the biosynthetic pathway in the host plant. Proc Natl Acad Sci USA 102: 9972–9977 10.1073/pnas.0500793102 15998742 PMC1174980

[35] Lombard J, Moreira D (2011) Origins and early evolution of the mevalonate pathway of isoprenoid biosynthesis in the three domains of life. Mol Biol Evol 28: 87–99 10.1093/molbev/msq177 20651049

[36] Gallegos MT, Schleif R, Bairoch A, Hofmann K, Ramos JL (1997) Arac/XylS family of transcriptional regulators. Microbiol Mol Biol Rev 61: 393–410. 9409145 10.1128/mmbr.61.4.393-410.1997 PMC232617

[37] Soisson SM, MacDougall-Shackleton B, Schleif R, Wolberger C (1997) Structural basis for ligand-regulated oligomerization of AraC. Science 276: 421–425. 9103202 10.1126/science.276.5311.421

[38] Kwon HJ, Bennik MH, Demple B, Ellenberger T (2000) Crystal structure of the *Escherichia coli* Rob transcription factor in complex with DNA. Nat Struct Biol 7: 424–430 10.1038/75213 10802742

[39] Lowden MJ, Skorupski K, Pellegrini M, Chiorazzo MG, Taylor RK, et al (2010) Structure of *Vibrio cholerae* ToxT reveals a mechanism for fatty acid regulation of virulence genes. Proc Natl Acad Sci USA 107: 2860–2865 10.1073/pnas.0915021107 20133655 PMC2840316

[40] Parmley JL, Hurst LD (2007) How common are intragene windows with KA>KS owing to purifying selection on synonymous mutations? J Mol Evol 64: 646–655 10.1007/s00239-006-0207-7 17557167

[41] Vereecke D, Cornelis K, Temmerman W, Jaziri M, Van Montagu M, et al (2002) Chromosomal locus that affects pathogenicity of *Rhodococcus fascians* . J Bacteriol 184: 1112–1120. 11807072 10.1128/jb.184.4.1112-1120.2002 PMC134788

[42] Bell KS, Philp JC, Aw DW, Christofi N (1998) The genus *Rhodococcus* . J Appl Microbiol 85: 195–210. 9750292 10.1046/j.1365-2672.1998.00525.x

[43] La Duc MT, Dekas A, Osman S, Moissl C, Newcombe D, et al (2007) Isolation and characterization of bacteria capable of tolerating the extreme conditions of clean room environments. Appl Environ Microbiol 73: 2600–2611 10.1128/AEM.03007-06 17308177 PMC1855582

[44] Konishi M, Nishi S, Fukuoka T, Kitamoto D, Watsuji T-O, et al. (2014) Deep-sea *Rhodococcus* sp. BS-15, Lacking the Phytopathogenic fas Genes, Produces a Novel Glucotriose Lipid Biosurfactant. Mar Biotechnol. doi:10.1007/s10126-014-9568-x. 10.1007/s10126-014-9568-x 24510374

[45] Yanai I, Wolf YI, Koonin EV (2002) Evolution of gene fusions: horizontal transfer versus independent events. Genome Biol 3: research0024. 12049665 10.1186/gb-2002-3-5-research0024 PMC115226

[46] Pasek S, Risler J-L, Brézellec P (2006) Gene fusion/fission is a major contributor to evolution of multi-domain bacterial proteins. Bioinformatics 22: 1418–1423 10.1093/bioinformatics/btl135 16601004

[47] Rajaonson S, Vandeputte OM, Vereecke D, Kiendrebeogo M, Ralambofetra E, et al (2011) Virulence quenching with a prenylated isoflavanone renders the Malagasy legume *Dalbergia pervillei* resistant to *Rhodococcus fascians* . Environ Microbiol 13: 1236–1252 10.1111/j.1462-2920.2011.02424.x 21332623

[48] Akiyoshi DE, Regier DA, Gordon MP (1987) Cytokinin production by *Agrobacterium* and *Pseudomonas* spp. J Bacteriol 169: 4242–4248. 3624204 10.1128/jb.169.9.4242-4248.1987 PMC213736

[49] Matsubara S, Armstrong DJ, Skoog F (1968) Cytokinins in tRNA of *Corynebacterium fascians* . Plant Physiology 43: 451–453. 16656786 10.1104/pp.43.3.451 PMC1086863

[50] Rathbone MP, Hall RH (1972) Concerning the presence of the cytokinin, N^6^-(Δ^2^-isopentnyl) adenine, in cultures of *Corynebacterium fascians* . Planta 108: 93–102 10.1007/BF00386072 24473817

[51] Einset JW, Skoog FK (1977) Isolation and identification of ribosyl–zeatin from transfer RNA of *Corynebacterium fascians* . Biochem Biophys Res Commun 79: 1117–1121 10.1016/0006-291X(77)91121-4 603646

[52] Miyawaki K, Tarkowski P, Matsumoto-Kitano M, Kato T, Sato S, et al (2006) Roles of Arabidopsis ATP/ADP isopentenyltransferases and tRNA isopentenyltransferases in cytokinin biosynthesis. Proc Natl Acad Sci USA 103: 16598–16603 10.1073/pnas.0603522103 17062755 PMC1637627

[53] Agris PF, Vendeix FAP, Graham WD (2007) tRNA’s Wobble Decoding of the Genome: 40 Years of Modification. J Mol Biol 366: 1–13 10.1016/j.jmb.2006.11.046 17187822

[54] Sprenger GA, Schörken U, Wiegert T, Grolle S, de Graaf AA, et al (1997) Identification of a thiamin-dependent synthase in *Escherichia coli* required for the formation of the 1-deoxy-D-xylulose 5-phosphate precursor to isoprenoids, thiamin, and pyridoxol. Proc Natl Acad Sci USA 94: 12857–12862. 9371765 10.1073/pnas.94.24.12857 PMC24228

[55] Lynch M (2006) Streamlining and simplification of microbial genome architecture. Annu Rev Microbiol 60: 327–349 10.1146/annurev.micro.60.080805.142300 16824010

[56] Takei K, Sakakibara H, Sugiyama T (2001) Identification of genes encoding adenylate isopentenyltransferase, a cytokinin biosynthesis enzyme, in *Arabidopsis thaliana* . J Biol Chem 276: 26405–26410 10.1074/jbc.M102130200 11313355

[57] Kuroha T, Tokunaga H, Kojima M, Ueda N, Ishida T, et al (2009) Functional analyses of *LONELY GUY* cytokinin-activating enzymes reveal the importance of the direct activation pathway in Arabidopsis. The Plant Cell 21: 3152–3169 10.1105/tpc.109.068676 19837870 PMC2782294

[58] Liu YG, Mitsukawa N, Oosumi T, Whittier RF (1995) Efficient isolation and mapping of *Arabidopsis thaliana* T-DNA insert junctions by thermal asymmetric interlaced PCR. Plant J 8: 457–463. 7550382 10.1046/j.1365-313x.1995.08030457.x

[59] Livak KJ, Schmittgen TD (2001) Analysis of relative gene expression data using real-time quantitative PCR and the 2^−ΔΔ*C*^T Method. Methods 25: 402–408 10.1006/meth.2001.1262 11846609

[60] Zerbino DR, Birney E (2008) Velvet: algorithms for de novo short read assembly using de Bruijn graphs. Genome Res 18: 821–829 10.1101/gr.074492.107 18349386 PMC2336801

[61] McLeod MP, Warren RL, Hsiao WWL, Araki N, Myhre M, et al (2006) The complete genome of *Rhodococcus* sp. RHA1 provides insights into a catabolic powerhouse. Proc Natl Acad Sci USA 103: 15582–15587 10.1073/pnas.0607048103 17030794 PMC1622865

[62] Rissman AI, Mau B, Biehl BS, Darling AE, Glasner JD, et al (2009) Reordering contigs of draft genomes using the Mauve aligner. Bioinformatics 25: 2071–2073 10.1093/bioinformatics/btp356 19515959 PMC2723005

[63] Altschul SF, Madden TL, Schäffer AA, Zhang J, Zhang Z, et al (1997) Gapped BLAST and PSI-BLAST: a new generation of protein database search programs. Nucleic Acids Res 25: 3389–3402. 9254694 10.1093/nar/25.17.3389 PMC146917

[64] Magrane M, Consortium U (2011) UniProt Knowledgebase: a hub of integrated protein data. Database (Oxford) 2011: bar009 10.1093/database/bar009 21447597 PMC3070428

[65] Eddy SR (2011) Accelerated Profile HMM Searches. PLoS Comp Biol 7: e1002195 10.1371/journal.pcbi.1002195 PMC3197634 22039361

[66] Darriba D, Taboada GL, Doallo R, Posada D (2011) ProtTest 3: fast selection of best-fit models of protein evolution. Bioinformatics 27: 1164–1165 10.1093/bioinformatics/btr088 21335321 PMC5215816

[67] Katoh K, Standley DM (2013) MAFFT Multiple Sequence Alignment Software Version 7: Improvements in Performance and Usability. Mol Biol Evol 30: 772–780 10.1093/molbev/mst010 23329690 PMC3603318

[68] Stamatakis A (2006) RAxML-VI-HPC: maximum likelihood-based phylogenetic analyses with thousands of taxa and mixed models. Bioinformatics 22: 2688–2690 10.1093/bioinformatics/btl446 16928733

[69] Sonnhammer ELL, Hollich V (2005) Scoredist: a simple and robust protein sequence distance estimator. BMC Bioinformatics 6: 108 10.1186/147121056108 15857510 PMC1131889

[70] Castresana J (2000) Selection of conserved blocks from multiple alignments for their use in phylogenetic analysis. Mol Biol Evol 17: 540–552. 10742046 10.1093/oxfordjournals.molbev.a026334

[71] Letunic I, Bork P (2011) Interactive Tree Of Life v2: online annotation and display of phylogenetic trees made easy. Nucleic Acids Res 39: W475–W478 10.1093/nar/gkr201 21470960 PMC3125724

[72] Quevillon E, Silventoinen V, Pillai S, Harte N, Mulder N, et al (2005) InterProScan: protein domains identifier. Nucleic Acids Res 33: W116–W120 10.1093/nar/gki442 15980438 PMC1160203

[73] Hunter S, Jones P, Mitchell A, Apweiler R, Attwood TK, et al (2012) InterPro in 2011: new developments in the family and domain prediction database. Nucleic Acids Res 40: D306–D312 10.1093/nar/gkr948 22096229 PMC3245097

[74] Crooks GE, Hon G, Chandonia J-M, Brenner SE (2004) WebLogo: a sequence logo generator. Genome Res 14: 1188–1190 10.1101/gr.849004 15173120 PMC419797

[75] Cole C, Barber JD, Barton GJ (2008) The Jpred 3 secondary structure prediction server. Nucleic Acids Res 36: W197–W201 10.1093/nar/gkn238 18463136 PMC2447793

[76] Waterhouse AM, Procter JB, Martin DMA, Clamp M, Barton GJ (2009) Jalview Version 2–a multiple sequence alignment editor and analysis workbench. Bioinformatics 25: 1189–1191 10.1093/bioinformatics/btp033 19151095 PMC2672624

[77] Zhang Z, Li J, Zhao X-Q, Wang J, Wong GK-S, et al (2006) KaKs_Calculator: calculating Ka and Ks through model selection and model averaging. Genomics Proteomics Bioinformatics 4: 259–263 10.1016/S16720229(07)600072 17531802 PMC5054075

[78] Zhang Z, Xiao J, Wu J, Zhang H, Liu G, et al (2012) ParaAT: a parallel tool for constructing multiple protein-coding DNA alignments. Biochem Biophys Res Commun 419: 779–781 10.1016/j.bbrc.2012.02.101 22390928

[79] Herbig A, Jager G, Battke F, Nieselt K (2012) GenomeRing: alignment visualization based on SuperGenome coordinates. Bioinformatics 28: i7–i15 10.1093/bioinformatics/bts217 22689781 PMC3371849

[80] Cole ST, Brosch R, Parkhill J, Garnier T, Churcher C, et al (1998) Deciphering the biology of *Mycobacterium tuberculosis* from the complete genome sequence. Nature 393: 537–544 10.1038/31159 9634230

[81] Bentley SD, Chater KF, Cerdeño-Tárraga A-M, Challis GL, Thomson NR, et al (2002) Complete genome sequence of the model actinomycete *Streptomyces coelicolor* A3(2). Nature 417: 141–147. 12000953 10.1038/417141a

[82] Cerdeño-Tárraga A-M, Efstratiou A, Dover LG, Holden MTG, Pallen M, et al (2003) The complete genome sequence and analysis of *Corynebacterium diphtheriae* NCTC13129. Nucleic Acids Res 31: 6516–6523. 14602910 10.1093/nar/gkg874 PMC275568

[83] Ishikawa J, Yamashita A, Mikami Y, Hoshino Y, Kurita H, et al (2004) The complete genomic sequence of *Nocardia farcinica* IFM 10152. Proc Natl Acad Sci USA 101: 14925–14930 10.1073/pnas.0406410101 15466710 PMC522048

[84] Na K-S, Kuroda A, Takiguchi N, Ikeda T, Ohtake H, et al (2005) Isolation and characterization of benzene-tolerant *Rhodococcus opacus* strains. Journal of Bioscience and Bioengineering 99: 378–382 10.1263/jbb.99.378 16233805

[85] Sekine M, Tanikawa S, Omata S, Saito M, Fujisawa T, et al (2006) Sequence analysis of three plasmids harboured in *Rhodococcus erythropolis* strain PR4. Environ Microbiol 8: 334–346 10.1111/j.1462-2920.2005.00899.x 16423019

[86] Chen H-P, Zhu S-H, Casabon I, Hallam SJ, Crocker FH, et al (2012) Genomic and transcriptomic studies of an RDX (hexahydro-1,3,5-trinitro-1,3,5-triazine)-degrading actinobacterium. Appl Environ Microbiol 78: 7798–7800 10.1128/AEM.02120-12 22923396 PMC3485706

[87] Supek F, Bošnjak M, Skunca N, Smuc T (2011) REVIGO summarizes and visualizes long lists of gene ontology terms. PLoS ONE 6: e21800 10.1371/journal.pone.0021800 21789182 PMC3138752

[88] Wu S, Zhu Z, Fu L, Niu B, Li W (2011) WebMGA: a customizable web server for fast metagenomic sequence analysis. BMC Genomics 12: 444 10.1186/1471-2164-12-444 21899761 PMC3180703

[89] R Core Team (2013) R: A Language and Environment for Statistical Computing. R Foundation for Statistical Computing. Available: http://www.R-project.org.

[90] Schneider CA, Rasband WS, Eliceiri KW (2012) NIH Image to ImageJ: 25 years of image analysis. Nat Meth 9: 671–675 10.1038/nmeth.2089 PMC5554542 22930834

[91] Vandeputte O, Oden S, Mol A, Vereecke D, Goethals K, et al (2005) Biosynthesis of auxin by the gram-positive phytopathogen *Rhodococcus fascians* is controlled by compounds specific to infected plant tissues. Appl Environ Microbiol 71: 1169–1177 10.1128/AEM.71.3.1169-1177.2005 15746315 PMC1065166

[92] Redig P, Schmülling T, Van Onckelen H (1996) Analysis of Cytokinin Metabolism in *ipt* Transgenic Tobacco by Liquid Chromatography-Tandem Mass Spectrometry. Plant Physiology 112: 141–148. 12226381 10.1104/pp.112.1.141 PMC157933

