# Supplementary material for: Analysis of Genome Sequences from Plant Pathogenic Rhodococcus Reveals Genetic Novelties in Virulence Loci
Source: PLoS One. 2014 Jul 10;9(7):e101996. doi: 10.1371/journal.pone.0101996 (PMC4092121; doi:10.1371/journal.pone.0101996)
Supplement: Table S2 — Reciprocal best BLAST hit analysis of pFiD188 against the genome assemblies of A25f and A21d2. *Locus identifiers from previously published pFID188 linear plasmid sequence; §Based on this study. (PDF) [file pone.0101996.s007.pdf]

Supplemental Table S2. Reciprocal best BLAST hit analysis of pFiD188 against the genome assemblies for A25f and A21d2

D188 locus IDs*	Annotated gene function[§]	D188 locus IDs	A25f locus ID	A21d2 locus ID	A25f %ID	A21d2 %ID
pFi_010	hypothetical protein	RFD188_04854		RFA21d2_02415		27.4%
pFi_037	putative transposase	RFD188_04878		RFA21d2_03325		88.7%
pFi_044	hypothetical protein	RFD188_04885		RFA21d2_02318		61.3%
pFi_045	hypothetical protein	RFD188_04886		RFA21d2_02317		56.3%
pFi_055	hypothetical protein	RFD188_04899	RFA25f_04154		87.1%	
pFi_056	putative aldo/ketoreductase family protein	RFD188_04900	RFA25f_04153		85.2%	
pFi_057	hypothetical protein	RFD188_04901	RFA25f_04152	RFA21d2_03351	88.3%	37.7%
pFi_058	putative integral membrane efflux protein of the major facilitator superfamily	RFD188_04902	RFA25f_04151		95.8%	
pFi_059	putative haloacid dehalogenase (HAD) superfamily hydrolase	RFD188_04903	RFA25f_04150		94.0%	
pFi_060	putative gamma-butyrolactone biosynthesis protein of the AfsA family	RFD188_04904	RFA25f_04149		97.8%	
pFi_061	putative MhpC-like hydrolase or acyltransferase	RFD188_04905	RFA25f_04148		90.5%	
pFi_062	putative drug exporter of the RND superfamily	RFD188_04906	RFA25f_04147		89.1%	
pFi_063	putative TetR family transcriptional regulator	RFD188_04907	RFA25f_04146		87.8%	
pFi_064 (attR)	LysR type transcriptional regulator	RFD188_04908	RFA25f_04137	RFA21d2_02316	94.5%	97.7%
pFi_065 (attX)	putative efflux protein of the LysE superfamily	RFD188_04909	RFA25f_04136	RFA21d2_02315	96.2%	97.1%
pFi_066 (*attA*)	putative argininosuccinate lyase	RFD188_04910	RFA25f_04135	RFA21d2_02314	98.6%	98.8%
pFi_067 (*attB*)	putative argininosuccinate synthase	RFD188_04911	RFA25f_04134	RFA21d2_02313	99.0%	99.0%
pFi_068 (*attC*)	putative folate-dependent phosphoribosylglycinamide formyltransferase	RFD188_04912	RFA25f_04133	RFA21d2_02312	97.9%	97.4%
pFi_069 (*attD*)	putative enoyl CoA hydratase/isomerase	RFD188_04913	RFA25f_04132	RFA21d2_02311	97.5%	99.3%
pFi_070 (*attE*)	putative beta-lactam synthetase	RFD188_04914	RFA25f_04131	RFA21d2_02310	96.1%	95.9%
pFi_071 (*attF*)	putative clavaminic acid synthetase	RFD188_04915	RFA25f_04130	RFA21d2_02309	97.2%	94.3%
pFi_072 (*attG*)	putative acetyl-CoA synthetase	RFD188_04916	RFA25f_04129	RFA21d2_02308	95.9%	96.1%
pFi_073 (*attH*)	putative bifunctional ornithine acetyl transferase/N-acetylglutamate synthase	RFD188_04917	RFA25f_04128	RFA21d2_02307	97.9%	93.0%
pFi_074 (*fasR*)	AraC type transcriptional regulator	RFD188_04918	RFA25f_04127	RFA21d2_02306	97.1%	75.0%
pFi_075	putative SAM-dependent methyltransferase	RFD188_04919	RFA25f_04126		98.2%	
pFi_076	putative SAM-dependent methyltransferase	RFD188_04920	RFA25f_04125		98.9%	
pFi_077 (*fasA*)	putative cytochrome P450 monooxygenase	RFD188_04921	RFA25f_04124		99.3%	
pFi_078 (*fasB*)	putative dual protein	RFD188_04922	RFA25f_04123		98.4%	
pFi_079 (*fasC*)	putative pyrimidine-binding transketolase	RFD188_04923	RFA25f_04122		98.1%	
pFi_080 (*fasD*)	isopentenyl transferase (IPT)	RFD188_04924	RFA25f_04121		95.2%	
pFi_081 (*fasE*)	cytokinin dehydrogenase/oxidase	RFD188_04925	RFA25f_04120		95.2%	
pFi_082 (*fasF*)	phosphoribohydrolase (LOG)	RFD188_04926	RFA25f_04119	RFA21d2_02304	97.5%	39.4%
pFi_083	putative dibenzothiophene desulfurization enzyme A	RFD188_04927	RFA25f_04118	RFA21d2_03196	99.1%	50.3%
pFi_084	putative acyltransferase PapA5	RFD188_04928	RFA25f_04117		91.5%	
pFi_094	putative peptidase family M23 protein	RFD188_04938	RFA25f_04558	RFA21d2_02435	44.0%	52.8%
pFi_095	hypothetical protein	RFD188_04939		RFA21d2_02434		39.7%
pFi_098	putative DNA translocase of the FtsK/SpoIIIE family	RFD188_04941		RFA21d2_02442		31.4%
pFi_108	putative (poly)peptide deformylase	RFD188_04951	RFA25f_04045		82.5%	
pFi_126	hypothetical protein	RFD188_04970		RFA21d2_02404		89.6%
pFi_129	hypothetical protein	RFD188_04973		RFA21d2_02400		93.8%

pFi_131	hypothetical protein	RFD188_04974		RFA21d2_02398		90.5%
pFi_144	gentisate 1,2-dioxygenase	RFD188_04986		RFA21d2_02231		31.0%
pFi_151	putative MerR family transcriptional regulator	RFD188_04993		RFA21d2_02407		60.0%
pFi_170	hypothetical protein	RFD188_05010		RFA21d2_02420		61.6%
pFi_183	hypothetical protein	RFD188_05023	RFA25f_03728		27.3%	
pFi_184	hypothetical protein	RFD188_05024		RFA21d2_02460		31.6%

*Locus identifiers from previously published pFID188 linear plasmid sequence; [§]Based on this study.
