# Supplementary material for: Analysis of Genome Sequences from Plant Pathogenic Rhodococcus Reveals Genetic Novelties in Virulence Loci
Source: PLoS One. 2014 Jul 10;9(7):e101996. doi: 10.1371/journal.pone.0101996 (PMC4092121; doi:10.1371/journal.pone.0101996)
Supplement: Table S3 — Homologs of enzymes of the methylerythritol phosphate pathway are present in all 20 isolates of Rhodococcus . *Locus; % identity; BLAST e-value. Column abbreviations: Deoxyxylulose 5-phosphate synthase (DXPS); DXP reductiosomerase (DXPR); isopentenyl diphosphate isomerase (IDI1); 4-diphosphocytidyl-2C-methyl-D-erythirol synthase (CMS); 4-(cytidine 5′-diphospho)-2-C-methyl-D-erythritol kinase (CMK); 2-C-methyl-D-erythritol 2,4-cyclodiphosphate synthase (MCS); hydroxy-2-methyl-2-(E)-butenyl 4-diphosphate synthase (HDS); hydroxy-2-methyl-2-(E)-butenyl 4-diphosphate reductase (HDR); No homologs of the MEP pathway were identified: 3-hydroxy-3-methylglutaryl-CoA synthase (HMGS; N. farcinia; YP_118423); 3-hydroxy-3-methylglutaryl-CoA reductase (HMGR; class I; N. farcinia; YP_118422); Mevalonate Kinase (MVK; N. farcinia; YP_118418); Phosphomevalonate kinase (PMK; N. farcinia; YP_118420); Mevalonate-5-decarboxylase (MDC; N. farcinia; YP_118419). YP_118418 identified homologs in isolates of sub-clades i and ii but e-values ranged from 6e-10 ∼ 3e-10 and CDSs are annotated as galactokinase. ∧Genbank ID number of sequence used for BLAST searches. (PDF) [file pone.0101996.s008.pdf]

Supplemental Table S3: Homologs of enzymes of the methylerythritol phosphate pathway are present in all 20 isolates of *Rhodococcus*

	DXPS*	DXPR*	CMS*	CMK*	MCS*	HDS*	IDS*	IDI1*
Nocardia farcinia^	YP_119953	YP_120333	YP_116642	YP_121117	YP_116643	YP_120331	YP_121011	YP_118189
GIC26	RFGIC26_02338<br>72.35; 0.0	RFGIC26_02145<br>74.13; 0.0	RFGIC26_00763<br>66.35; 2e-82	RFGIC26_03805<br>75.90; 5e-161	RFGIC26_00764<br>81.82; 1e-83	RFGIC26_02147<br>87.34; 0.0	RFGIC26_01206<br>84.48; 0.0	RFGIC26_02627<br>43.40; 2e-29
GIC36	RFGIC36_02362<br>72.35; 0.0	RFGIC36_02108<br>73.87; 0.0	RFGIC36_00754<br>65.71; 4e-81	RFGIC36_03898<br>76.22; 1e-161	RFGIC36_00755<br>82.47; 1e-83	RFGIC36_02110<br>87.34; 0.0	RFGIC36_01284<br>84.18; 0.0	RFGIC36_02651<br>43.40; 2e-29
05-561-1	RF05561_02716<br>72.31; 0.0	RF05561_02520<br>74.13; 0.0	RF05561_00754<br>65.40; 2e-80	RF05561_04429<br>75.90; 7e-161	RF05561_00755<br>82.47; 1e-83	RF05561_02522<br>87.34; 0.0	RF05561_01245<br>84.18; 0.0	RF05561_02957<br>43.40; 2e-29
LMG3605	RF3605_02508<br>72.51; 0.0	RF3605_02175<br>73.87; 0.0	RF3605_04159<br>65.88; 1e-81	RF3605_03773<br>76.22; 2e-161	RF3605_04158<br>82.47; 1e-83	RF3605_02177<br>87.34; 0.0	RF3605_01317<br>84.18; 0.0	RF3605_02797<br>43.40; 1e-29
D188	RFD188_02269<br>72.51; 0.0	RFD188_02074<br>73.87; 0.0	RFD188_00725<br>65.88; 1e-81	RFD188_03538<br>76.22; 3e-161	RFD188_00726<br>82.47; 1e-83	RFD188_02076<br>87.34; 0.0	RFD188_01214<br>84.18; 0.0	RFD188_02558<br>43.40; 1e-29
LMG3602	RF3602_03412<br>72.51; 0.0	RF3602_03060<br>74.40; 0.0	RF3602_00204<br>65.71; 2e-81	RF3602_04805<br>76.22; 1e-161	RF3602_00203<br>82.47; 1e-83	RF3602_03062<br>87.34; 0.0	RF3602_02266<br>84.18; 0.0	RF3602_03698<br>42.77; 3e-29
LMG3616	RF3616_02691<br>72.51; 0.0	RF3616_02316<br>73.87; 0.0	RF3616_00833<br>66.35; 2e-82	RF3616_03691<br>76.22; 2e-161	RF3616_00834<br>82.47; 1e-83	RF3616_02318<br>87.86; 0.0	RF3616_01325<br>84.18; 0.0	RF3616_02978<br>42.77; 3e-29
A3b	RFA3b_02032<br>72.35; 0.0	RFA3b_01766<br>74.13; 0.0	RFA3b_00611<br>66.35; 3e-82	RFA3b_03832<br>76.22; 3e-161	RFA3b_00612<br>82.47; 1e-83	RFA3b_01768<br>87.08; 0.0	RFA3b_01082<br>83.88; 0.0	RFA3b_02350<br>42.77; 3e-29
**LMG3623**	RF3623_02067<br>72.51; 0.0	RF3623_01470<br>73.87; 0.0	RF3623_00294<br>65.88; 2e-81	RF3623_03568<br>76.22; 2e-161	RF3623_00295<br>82.47; 1e-83	RF3623_01472<br>87.34; 0.0	RF3623_04267<br>84.18; 0.0	RF3623_02356<br>42.77; 3e-29
A78	RFA78_02580<br>72.35; 0.0	RFA78_02388<br>73.87; 0.0	RFA78_00866<br>65.71; 7e-82	RFA78_03918<br>76.22; 4e-161	RFA78_00867<br>81.82; 4e-83	RFA78_02390<br>87.34; 0.0	RFA78_01369<br>84.18; 0.0	RFA78_02867<br>43.40; 2e-29
A21d2	RFA21d2_01886<br>72.86; 0.0	RFA21d2_01613<br>74.13; 0.0	RFA21d2_00584<br>65.88; 2e-81	RFA21d2_04036<br>75.42; 5e-158	RFA21d2_00585<br>80.79; 1e-82	RFA21d2_01615<br>87.60; 0.0	RFA21d2_01116<br>84.48; 0.0	RFA21d2_02183<br>40.61; 6e-26
04-516	RF04516_02331<br>72.70; 0.0	RF04516_02140<br>73.87; 4e-180	RF04516_05280<br>66.35; 3e-82	RF04516_01001<br>75.42; 5e-158	RF04516_05281<br>80.79; 1e-82	RF04516_02142<br>87.60; 0.0	RF04516_00530<br>84.48; 0.0	RF04516_02628<br>40.61; 6e-26
A25f	RFA25f_02996<br>73.02; 0.0	RFA25f_02802<br>74.13; 0.0	RFA25f_00482<br>65.88; 3e-82	RFA25f_02465<br>75.08; 2e-157	RFA25f_00483<br>80.79; 1e-82	RFA25f_02804<br>87.60; 0.0	RFA25f_01062<br>84.48; 0.0	RFA25f_03300<br>40.61; 1e-25
LMG3625	RF3625_04912<br>73.02; 0.0	RF3625_03926<br>73.87; 3e-180	RF3625_02426<br>66.35; 5e-83	RF3625_04242<br>74.91; 2e-151	RF3625_02427<br>80.79; 1e-82	RF3625_03928<br>87.34; 0.0	RF3625_02964<br>83.88; 0.0	RF3625_05210<br>40.00; 5e-25
05-339-1	RF05339_02297<br>72.83; 0.0	RF05339_02107<br>74.06; 0.0	RF05339_00278<br>65.07; 1e-77	RF05339_04009<br>75.42; 3e-157	RF05339_00277<br>79.87; 4e-83	RF05339_02109<br>87.60; 0.0	RF05339_01291<br>84.18; 0.0	RF05339_02591<br>41.21; 3e-26
A76	RFA76_02494<br>72.83; 0.0	RFA76_02302<br>74.40; 0.0	RFA76_05363<br>65.14; 2e-85	RFA76_03930<br>75.42; 3e-157	RFA76_05364<br>79.87; 3e-83	RFA76_02304<br>87.60; 0.0	RFA76_01654<br>84.18; 0.0	RFA76_02795<br>40.34; 4e-27
A44a	RFA44a_02292<br>74.13; 0.0	RFA44a_02099<br>74.40; 0.0	RFA44a_00645<br>65.47; 4e-88	RFA44a_05247<br>76.55; 5e-162	RFA44a_00646<br>81.46; 5e-82	RFA44a_02101<br>89.38; 0.0	RFA44a_01139<br>83.28; 0.0	RFA44a_01395<br>41.21; 4e-26
02-815	RF02815_02526<br>74.13; 0.0	RF02815_02333<br>74.40; 0.0	RF02815_01093<br>64.57; 4e-86	RF02815_04653<br>76.55; 6e-162	RF02815_01094<br>81.46; 5e-82	RF02815_02335<br>89.38; 0.0	RF02815_01297<br>83.28; 0.0	RF02815_02069<br>40.61; 2e-25
02-816c	RF02816c_02438<br>74.13; 0.0	RF02816c_02246<br>74.40; 0.0	RF02816c_00772<br>65.02; 6e-87	RF02816c_04485<br>76.55; 6e-162	RF02816c_00773<br>81.46; 5e-82	RF02816c_02248<br>89.38; 0.0	RF02816c_01507<br>83.28; 0.0	RF02816c_01700<br>40.61; 2e-25
A73a	RFA73a_02592<br>74.13; 0.0	RFA73a_02400<br>74.40; 0.0	RFA73a_00558<br>64.57; 4e-86	RFA73a_01123<br>76.55; 5e-162	RFA73a_00559<br>81.46; 5e-82	RFA73a_02402<br>89.38; 0.0	RFA73a_01858<br>83.28; 0.0	RFA73a_01567<br>40.61; 2e-25

*Locus; % identity; BLAST e-value. Column abbreviations: Deoxyxylulose 5-phosphate synthase (DXPS); DXP reductiosomerase (DXPR); isopentenyl diphosphate isomerase (IDI1); 4-diphosphocytidyl-2C-methyl-D-erythirol synthase (CMS); 4-(cytidine 5'-diphospho)-2-C-methyl-D-

erythritol kinase (CMK); 2-C-methyl-D-erythritol 2,4-cyclodiphosphate synthase (MCS); hydroxy-2-methyl-2-(E)-butenyl 4-diphosphate synthase (HDS); hydroxy-2-methyl-2-(E)-butenyl 4-diphosphate reductase (HDR); No homologs of the MEP pathway were identified: 3-hydroxy-3-methylglutaryl-CoA synthase (HMGS; *N. farcinia*; YP_118423); 3-hydroxy-3-methylglutaryl-CoA reductase (HMGR; class I; *N. farcinia*; YP_118422); Mevalonate Kinase (MVK; *N. farcinia*; YP_118418); Phosphomevalonate kinase (PMK; *N. farcinia*; YP_118420); Mevalonate-5-decarboxylase (MDC; *N. farcinia*; YP_118419). YP_118418 identified homologs in isolates of sub-clades i and ii but e-values ranged from 6e-10 ~ 3e-10 and CDSs are annotated as galactokinase. ^Genbank ID number of sequence used for BLAST searches.
