# Supplementary material for: Analysis of Genome Sequences from Plant Pathogenic Rhodococcus Reveals Genetic Novelties in Virulence Loci
Source: PLoS One. 2014 Jul 10;9(7):e101996. doi: 10.1371/journal.pone.0101996 (PMC4092121; doi:10.1371/journal.pone.0101996)
Supplement: Table S4 — Cytokinin types that were profiled from D188. (PDF) [file pone.0101996.s009.pdf]

Supplemental Table S4: Cytokinin types that were profiled from D188

Cytokinin Type	Abbreviation	Detected (Y/N)
Isopentenyladenine	iP	Y
2-methylthio isopentenyladenine	MS-iP	N
isopentenyladenine glucoside	iP-G	N
isopentenyladenosine	iPA	Y
2-methylthio isopentenyladenosine	MS-iPA	N
isopentenyladenine ribotide	iPAMP	Y
cis-zeatin	cZ	Y
cis-zeatin riboside	cZR	Y
2-methylthio-cis-zeatin	2MeScZ	Y
2-methylthio-cis-zeatin riboside	2MeScZR	Y
trans-zeatin	tZ	N
trans-zeatin riboside	tZR	N
zeatin N-glucoside	ZNG	N
trans-zeatin ribotide	tZRMP	Y
Dihydrozeatin	DHZ	N
dihydrozeatin riboside	DHZR	N
dihydro zeatin N-glucoside	DHZNG	N
dihydrozeatin riboside phosphate	DHZR-MP	N
ortho-topolin	o-Top	N
meta-topolin	m-Top	N
para-topolin	p-Top	N
ortho-topolin riboside	o-TopR	N
meta-topolin riboside	m-TopR	N
para-topolin riboside	p-TopR	N
ortho-topolin glucoside	o-TopG	N
meta-topolin glucoside	m-TopG	N
para-topolin glucoside	p-TopG	N
6-benzylaminopurine	BAP	N
6-benzylaminopurine riboside	BAPR	N
3-glucoside	BAP3G	N
7-glucoside	BAP7G	N
9-glucoside	BAP9G	N
