# Supplementary material for: Analysis of Genome Sequences from Plant Pathogenic Rhodococcus Reveals Genetic Novelties in Virulence Loci
Source: PLoS One. 2014 Jul 10;9(7):e101996. doi: 10.1371/journal.pone.0101996 (PMC4092121; doi:10.1371/journal.pone.0101996)
Supplement: Table S5 — Sequences of oligonucleotides used in this study. (PDF) [file pone.0101996.s010.pdf]

Supplemental Table S5: Oligonucleotide sequences

Target Sequence	Purpose	Oligonucleotide sequence (5'-3')
16s rDNA	*Rhodococcus*-specific positive control	TACGGAGAGTTTGATCCTGGC (top)
		AGAAAGGAGGTGATCCAGCCG (bottom)
	qRT-PCR housekeeping primers	CCAAGGCGACGACGGGTAGC (top)
		TCGCTGCATCAGGCTTCCGC (bottom)
pFi_013	Linear plasmid R1 region	ATGGCAATATCAAATTCCCTC (top)
		TCATGCGGGTGCTACCTCTTC (bottom)
pFi_070 and RFA21d2_02310 (*attE*)	qRT-PCR	ACTCCTCGCCTCCTACTTGTGG (top)
		GCGGAGGTAGTACTTTTCGATGC (bottom)
pFi_080 (*fasD*)	*fasD* (full length)	CAAAAAAGCAGGCTCCATGAAGGAATCAACCATGGCA (top)
		GAAAGCTGGGTGTCTGGCGGTCACACCTGGGGC (bottom)
	qRT-PCR	TGACCGGCTGATAAAAGTACTGACC (top)
		ACCAGTCGAGTACACAGTCCAGACC (bottom)
RFA21d2_02304 (*fasD-F* fusion)	A21d2_02304 (full length)	CAAAAAAGCAGGCTCCATGTCGCCTTTGTACTCG (top)
		GAAAGCTGGGTGGTCGTCATTGCCATCCTG (bottom)
	RT-PCR	TGATAGTCACCGGTGATC (top)
		TTGCCTAGTTCTACTGCC (bottom)
RFA21d2_02303	A21d2_02303 (full length)	CAAAAAAGCAGGCTCCATGACGACTGA/AGGTTCA (top)
		GAAAGCTGGGTGCTATCTGGCTCTATCGTC (bottom)
	RT-PCR	AGCTTTGAGGTAATTCCTACT (bottom)
RFA21d2_02305	A21d2_02305 (full length)	CAAAAAAGCAGGCTCCATGAGGGACGGGCTGCAA (top)
		GAAAGCTGGGTGCTTCTCGACCTTGTTGGA (bottom)
	RT-PCR	AGATGCCACTTGTGCCTTCCG (top)
Contig 21 (A21d2)	TAIL-PCR (two nested sets for left (GSL1-3) and right side (GSR1-3) of contig 21)	CGCTGACGGCCCAGAGCACG (GSL1)
		CCTCGATCGTGCAGAAGCTG (GSL2)
		CAAGTGGAGATTGTTCTTCG (GSL3)
		GAACTCGCAGCGTCGATATG (GSR1)
		GATGGACAAGGACACACGGG (GSR2)
		CGACGTTGATCTGGGTACCG (GSR3)
