# Supplementary material for: Analysis of Genome Sequences from Plant Pathogenic Rhodococcus Reveals Genetic Novelties in Virulence Loci
Source: PLoS One. 2014 Jul 10;9(7):e101996. doi: 10.1371/journal.pone.0101996 (PMC4092121; doi:10.1371/journal.pone.0101996)
Supplement: Table S6 — Parent ions and diagnostic transitions used in multiple reactant monitoring (MRM) for the analysis of cytokinins by ACQUITY TQP UPLC-MS/MS. (PDF) [file pone.0101996.s011.pdf]

Supplemental Table S6: Parent ions and diagnostic transitions used in multiple reactant monitoring (MRM) for the analysis of cytokinins by ACQUITY TQP UPLC-MS/MS

Compound	Parent ion	Diagnostic transition MRM
iP	204	204 → 136
[$^2H_6$]iP	210	210 → 137
iPA	336	336 → 204
[$^2H_6$][9R]iP	342	342 → 210
Z	220	220 → 136
DHZ	222	222 → 136
[$^2H_5$]DHZ	225	225 → 136
ZR	352	352 → 220
[$^2H_5$][9R]DHZ	354	354 → 222
MS-Z	266	266 → 182
[$^2H_5$]MSZ	271	271 → 182/183
MS-ZR	398	398 → 266
[$^2H_5$]MSZR	403	403 → 271
